# Structured Catalysts for Continuous Biphasic Furfural Synthesis from Biorefinery Feedstock

**DOI:** 10.1021/acssuschemeng.5c05251

**Published:** 2025-10-14

**Authors:** Adarsh Patil, Afnan Ahmad, Maria Fernanda Neira D’Angelo

**Affiliations:** Chemical Reactor Engineering Laboratory, Sustainable Process Engineering, Department of Chemical Engineering and Chemistry, 3169 Eindhoven University of Technology, Eindhoven 5600 MB, The Netherlands

**Keywords:** furfural, xylose, structured catalysts, biorefinery, biphasic

## Abstract

Sustainable chemicals from lignocellulosic biomass can accelerate material transition. Heterogeneous catalysts provide a superior alternative to homogeneous catalysts for xylose dehydration to furfural, a promising platform chemical. This work uses 3D open-cell aluminum foam structures as catalyst support for the biphasic furfural synthesis from biorefinery hydrolysate (obtained via birch pretreatment). 3D open-cell aluminum foams were coated with commercial TiO_2_. The foams were dip-coated in a solid slurry consisting of TiO_2_ dispersed in water and binders. Coatings were found to be reproducible and mechanically stable. Catalytic activity testing of the TiO_2_ foams showed ∼60–70% furfural selectivity at near-complete xylose conversion in the range of 170–190 °C. The enhanced mass transport properties of foams minimized the formation of insoluble humin species in the aqueous phase. Employing *sec*-butylphenol (SBP) as the organic extractant enables long-term operation for at least 36 h. This is due to the coextraction of furfural and 5-hydroxymethylfurfural (obtained from glucose in the feed). Varying foam thickness while maintaining constant mass of washcoat showed absence of both external and internal mass transfer limitations. Finally, performing xylose dehydration at 190 °C using TiO_2_-coated foams enabled a remarkably high furfural productivity of 5.8 × 10^–2^ g_furfural_ g_cat_^–1^ min^–1^, over an order of magnitude greater than the highest reported in the literature.

## Graphical abstract

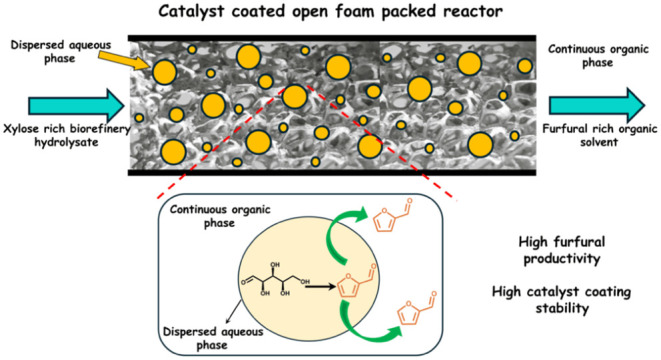

## 1 Introduction

The chemical industry contributes approximately 5.7 trillion US $ to global GDP while supporting 120 million jobs.^1^ Despite its immense contributions to the modern lifestyle, it bears negative environmental consequences. Utilizing renewable biomass such as lignocellulose, comprising cellulose, hemicellulose, and lignin, can limit these issues. Dehydration of hemicellulosic sugars in acidic environment yields furfural, an attractive platform molecule in the production of chemicals and materials.^2–4^ Current industrial processes (Huaxia/Westpro) are still limited to ∼50% furfural yield^5^ of the theoretical pentosan content while consuming large amounts of steam.^6^ Several degradation pathways originating from xylose, intermediates, and furfural, resulting in high molecular weight species, commonly referred to as humins limit furfural yields.^7,8^

Low furfural yields, poor economics, and biomass procurement challenges have caused the closure of several plants producing furfural from biomass in a single step.^6,9,10^ Recent works propose biomass fractionation to obtain three different aqueous streams, commonly known as hydrolysates, as the first step. Consequently, the separate conversion of hemicellulosic streams to furfural and cellulosic streams to ethanol maximizes biomass valorization via a two-step strategy proposed by Bao et al.^11^ Inspired by the traditional Quaker Oats’ process, where furfural is isolated in situ from the aqueous phase to increase its yield, the more recent biphasic furfural production process uses water-immiscible organic solvents to extract furfural in situ for the same purpose. Another strategy widely adopted in the literature to successfully obtain high furfural yields is the use of a miscible organic cosolvent to prevent furfural degradation upon its formation. While furfural yields above 80% have been reported for both the miscible^12–14^ and immiscible solvent systems, humin formation cannot be entirely prevented. The latter scenario simplifies downstream furfural purification. Numerous water-immiscible solvents such as toluene,^15^ 2-butanol,^16^ ethyl acetate,^17^ methyl isobutyl ketone (MIBK),^18^ *o-sec*-butyl phenol (SBP),^19^ and deep-eutectic solvents (DES)^20^ have been used for furfural synthesis. While using immiscible solvents enhances the E-factor for furfural production by minimizing humin formation, the amount of furfural produced per mass of hazardous substance decreases (see Table S1 for detailed information). Additionally, many of the aforementioned solvents, except for MIBK, fail to pass the SH&E criteria laid out by CHEM21 solvent selection guidelines.^21^ Leveraging a favorable E-factor using immiscible solvents, recent research has focused on combining it with heterogeneous solid acid catalysts to achieve furfural yields that are on par with those of the mineral and organic acids. Examples such as β-zeolite,^22^ H-ZSM5,^23^ γ-alumina, sulfonated zirconia/titania,^24,25^ H^+^ ion-exchange resins,^26,27^ and mixed metal oxides such as WO_3_/ZrO_2_ ^28^ have been reported to catalyze xylose dehydration to furfural. However, the combined performance of solid acid catalysts in a biphasic furfural production process from relevant biorefinery hydrolysates (see Table S1) has received limited attention.

In addition, Papaioannou et al.^15^ and Guo et al.^18^ have shown that fast liquid–liquid mass transfer rates are important to ensure effective furfural extraction at high reaction temperatures (ca. 160 to 190 °C) using micro/milli-reactors. Operating within these hydrodynamic regimes can enable high feed throughput at low residence times (i.e., <3 min) with minimal humins formation. These reactors suffer from high pressure drop and complex flow distribution control while scaling or numbering up.^29^ Using open-cell foam structures, characterized by high specific external surface area (900–2000 m^2^ m^–3^), high bed voidage (∼0.84–0.95), and interconnectivity between individual cells, can provide optimal interphase transport properties, similar to those in micro/milli-reactors, at significantly lower pressure drop across the reactor length.^30,31^ Furthermore, applying a thin catalyst coating on these structures can leverage transport advantages with optimal heterogeneous catalytic activity to improve furfural selectivity through in situ liquid–liquid extraction in biphasic systems. Our previous study demonstrated the potential of using Amberlyst-like coated foam structures as catalyst support with model xylose feed for improved catalytic activity and transport properties. However, at temperatures above 170 °C, we observed transport limitations in the resin layers, poor extraction efficiency, and humin deposition on the catalyst. So far, the use of 3D coated catalytic foam structures to convert real sugar hydrolysate mixture, while harnessing their transport properties for high yields and catalyst/reactor stability, has not been reported yet.

This work aims to demonstrate the benefits of combining high catalytic activity with rapid mass transfer rates using a foam structure coated with commercially available TiO_2_, a porous solid acid material with better intraparticle transport properties than acid resins and, based on batch-wise catalyst screenings, offers better selectivity vs conversion compared to other porous solid acids for this reaction. The coated foams were then used for the biphasic synthesis of furfural from a biorefinery hydrolysate obtained from birch pretreatment^32^ in flow conditions. To evaluate this concept, we have developed a coating procedure to deposit a catalytically active and stable TiO_2_ powder on the surface of commercially available aluminum foams. In this study, we discuss the performance of the TiO_2_-coated foam structures under different conditions (i.e., 160–190 °C, 1–5 min residence time, and different hydrophobic organic solvent medium). Moreover, we explore the effect of varying foam thickness and feed concentrations on xylose conversion and furfural selectivity. Additionally, we elucidate the effect of different substances present in the hydrolysate feed stream on process performance. The work concludes with a long-term stability testing of the coated foam structures and a discussion highlighting the benefits and limitations of this concept. Based on the experimental evidence and transport calculations provided in Section 3, we believe this work can be used as a framework for producing furfural from biorefinery hydrolysates.

## 2 Experimental Section

Catalytic foams were prepared by coating aluminum foams, which were first pretreated, then dip-coated in a solid slurry (consisting of the powdered catalyst and binders), followed by drying and calcination in an oven. Details regarding the foam preparation protocol can be found in Subsection 2.1 of the SI.

Bare catalyst powder (commercial TiO_2_, Degussa *p*-25, procured from Sigma-Aldrich and consisting of 80–20% mix of anatase and rutile phases, respectively) and its coated counterpart, simulated by calcining the binder and catalyst suspension, were characterized using XRD, BET, and NH_3_-TPD techniques. The thickness of foam coatings was determined using SEM and benchmarked using theoretical calculations based on TiO_2_ density and mass of coatings obtained after calcination. Details regarding the respective characterization techniques can be found in Sections 2.4 and 2.5 of the SI.

The coated foams were then loaded in a tubular flow reactor, which was placed in a downflow configuration in a circulating air oven. Aqueous hydrolysate stream and the organic solvent were flowed cocurrently through the reactor using a Knauer Smartline 1000 HPLC pump and a Teledyne ISCO 500D syringe pump, respectively. Details of reactor dimensions and the auxiliary equipments used are described in Section 2.2 of the SI. Samples collected after regular intervals were filtered and analyzed using HPLC and GC, outlined in Section 2.3 of the SI, which were then used to determine xylose conversion and furfural selectivity (according to eqs S2–S7).

The aqueous hydrolysate was provided by TNO (Nederlandse Organisatie voor Toegepast Natuurwetenschappelijk Onderzoek), Petten, The Netherlands. Fractionation of birch wood chips using an acetone organosolv process, as described elsewhere,^33^ produced the hydrolysate used as the feed in this work. For a typical activity test, the hydrolysate consists of 5.7 wt % xylose, 0.6 wt % arabinose, 0.8 wt % glucose, <0.1 wt % C6 sugars (fructose, mannose, and galactose, cumulatively), and <100 ppm furfural and 5-hydroxymethylfurfural (HMF). It should be noted that the overlapping of glucose and arabinose peaks while using the HPLC analytical method prevented the reliable determination of HMF selectivity from C6 sugars in the feed.

## 3 Results and Discussion

### 3.1 Foam Coating and Catalyst Characterization

Figure 1 shows visuals of the uncoated and TiO_2_-coated foam (40 PPI). SEM pictures show a plain and fracture-free surface of the coatings. Placing an orthogonal cut through the coating near the strut structure in the foam enables us to visualize the coating thickness, as shown in Figure 1D.

**Figure 1:**
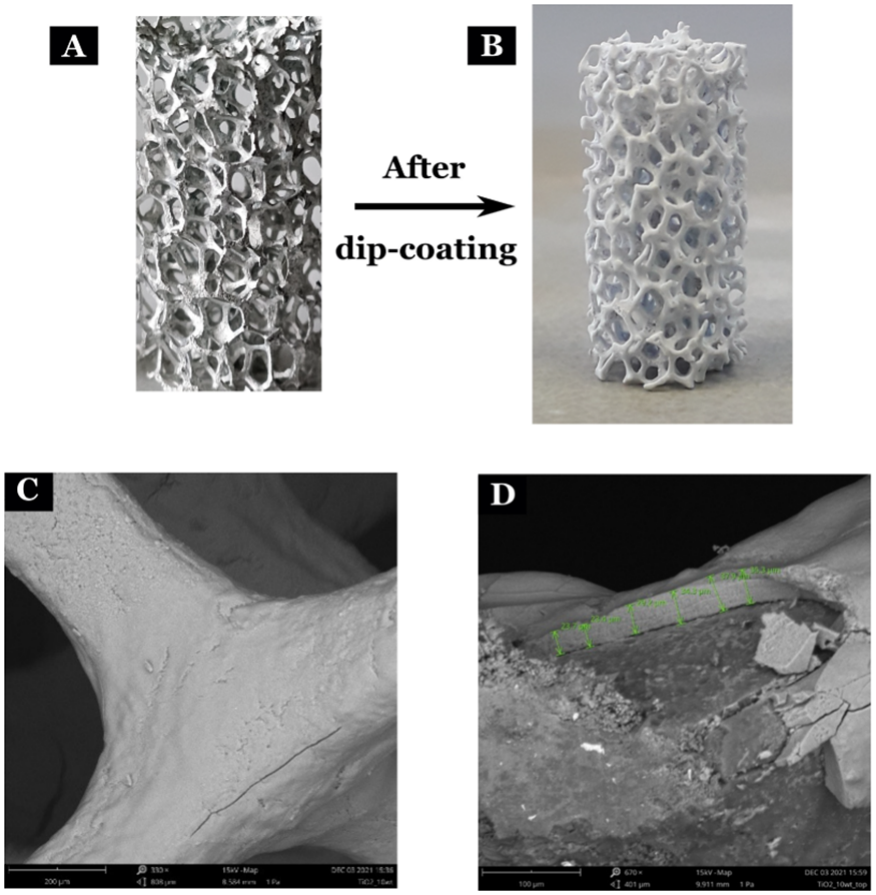
Visual and SEM pictures of open-cell foam structures. A: Bare foam; B: TiO_2_-coated foam; C: SEM image of the coated surface; D: SEM image showing coating thickness near a strut of the foam structure.

Table S2 depicts the mass uptake of a foam structure (40 PPI) after a number of dip-coatings in the TiO_2_ solids slurry and prior to calcination. The average mass of TiO_2_ coating obtained after 4 dip-coating repetitions (entries 2–5 in Table S2) followed by calcination is 118.9 ± 7.3 mg. These results demonstrate the reproducibility of the washcoating procedure used in this work. Moreover, the mass loss after calcination typically ranges from 26 to 34%, similar to the theoretical weight composition of the binders in the solids slurry, i.e., 33%. Table 1 depicts the washcoat mass uptake for the case of 131.2 mg coated TiO_2_ foam after each dip-coating step. Approximately 32–34 mg of coating is achieved after each dip-coating and drying for four repetitions. A comparison between the theoretical thickness, calculated using the method described in Section 2.4.1 of the SI, and SEM shows good agreement between the two values. Besides, SEM images show a smooth surface of the obtained coating. Passing an aqueous flow rate of 1 mL min^–1^ (see Section 2.4.2 of the SI for method description) through the coated foam led to a maximum 1.2 wt % loss of the catalyst coating, thereby demonstrating an excellent mechanical stability and resistance to erosion of the layer.

**Table 1:** Mass Uptake of an Open-Cell Aluminum Foam (Pore Density = 40 PPI) after Each Dip-Coating in a 10 wt % TiO_2_ and 2 + 3 wt % (PVA+PEG) in Water Slurry

No. of dip-coats	Mass uptake after calcination, mg	SEM thickness, μm	Theoretical thickness, μm
1	32.4	10–15	6.29
2	64	15–20	15.36
3	98.9	15–20	21.23
4	131.2	20–25	28.52

Figure 2 shows the XRD patterns and NH_3_-TPD, while Table 2 depicts the textural properties and total acid content of the solid catalysts upon thermal treatment before and after addition of the binders (PVA and PEG), albeit prior to dip-coating of the aluminum foams. The crystallinity of the coated TiO_2_ was corroborated with commercial Degussa *p*-25 (JCPDS 00-021-1272), showing an identical XRD pattern. Furthermore, no significant differences in the full width at half-maximum (fwhm) and intensity for any of the characteristic peaks were observed, thus confirming identical crystallite sizes of the dominant anatase phase present in TiO_2_. No shift in characteristic peaks indicates that binder removal through thermal treatment does not strain the TiO_2_-coated material. The available surface area for adsorption and the pore volume remain unaffected, suggesting that the coated material has not undergone physical changes during the coating procedure. Figure 2B shows the NH_3_-TPD curves for TiO_2_ before and after foam coating. A mild shift toward weaker acid sites is observed in the sample with binders, suggesting that some residual binder might remain, even after calcination. However, this is very likely minimal since calcination treatment takes place at 450 °C, well above the decomposition temperatures of the binders (around 300 °C), the total acid sites content (in $\mathrm{mmol}g_{\mathrm{cat}}^{-1}$ ) listed in Table 2 is the same with and without the addition of binder to the catalyst slurry. Thus, the dip-coating procedure does not significantly alter the surface properties of TiO_2_.

**Figure 2:**
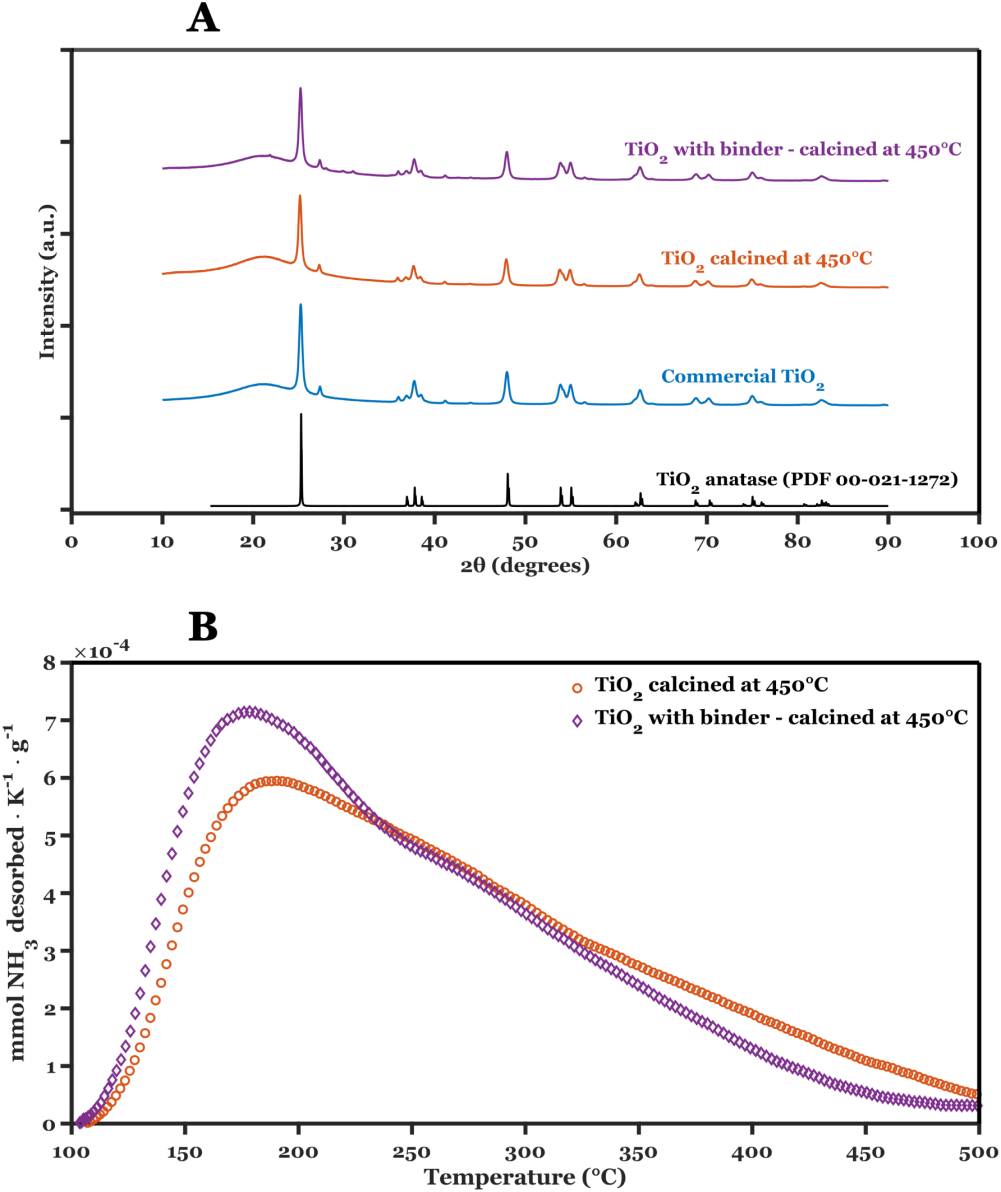
Characterization results comparing TiO_2_ powder before and after addition of binders (3 wt % PVA and 2 wt % PEG). A: XRD patterns, B: NH_3_-TPD results.

**Table 2:** Physicochemical Properties for TiO_2_ Powder with and without Binder

Material	*S*_BET_ ^a^ (m^2^ g^–1^)	Pore volume (cm^3^ g^–1^)	Total acidity (mmol g^–1^)
TiO_2_ powder calcined at 450 °C	53.1	0.18	0.120
TiO_2_ powder and binder slurry calcined at 450 °C	53.0	0.20	0.123

a Surface area by BET.

### 3.2 Catalytic Testing

#### 3.2.1 Effect of Temperature

Following the coating of foams with TiO_2_, their respective catalytic performance was investigated. Figure 3 shows xylose conversion and furfural selectivity obtained at different reaction temperatures using a fixed volumetric flow rate of the hydrolysate feed and organic solvent. The increased xylose conversion with temperature follows the expected Arrhenius dependence of reaction kinetics. On the other hand, furfural selectivity first increases and then plateaus at temperatures above 170 °C. Hence, increasing reaction temperature within the range explored in this study results in better furfural yields and productivity per mass of catalyst (i.e., from 11% to 48% with an increase in reaction temperature from 160 to 190 °C, respectively). Assuming that furfural extraction is not limiting (i.e., furfural extraction rates are significantly faster than furfural formation and degradation), a monotonic increase in furfural selectivity with temperature would be anticipated. However, furfural selectivity reaches a plateau. In this experimental set (performed at fixed liquid flow rates), the overall liquid–liquid mass transfer coefficient (*k*_*l*_ *a*) for furfural extraction from the aqueous media into the organic solvent is assumed to be constant. The furfural partition coefficient (denoted by *m*, and defined according to eq 2) is strongly affected by temperature. In our case, the partition coefficient of SBP follows an exponential decaying trend with operating temperature (see Figure S22). This results in a decrease in driving force and thus lower furfural extraction rates, as shown by eq 1, upon increasing operating temperature. In contrast, furfural degradation rates increase with temperature following the Arrhenius law. Therefore, furfural selectivity stagnates beyond 170–190 °C because degradation rates surpass extraction rates, which decrease with temperature due to a reduced *m*.

1 $$\mathrm{Furfural}\;\mathrm{extraction}\;\mathrm{rate}=k_{l}a\left(C_{furfural,aq}-\frac{C_{furfural,org}}{m}\right)$$

2 $$m=\frac{C_{org,furfural}}{C_{aq,furfural}}$$

**Figure 3:**
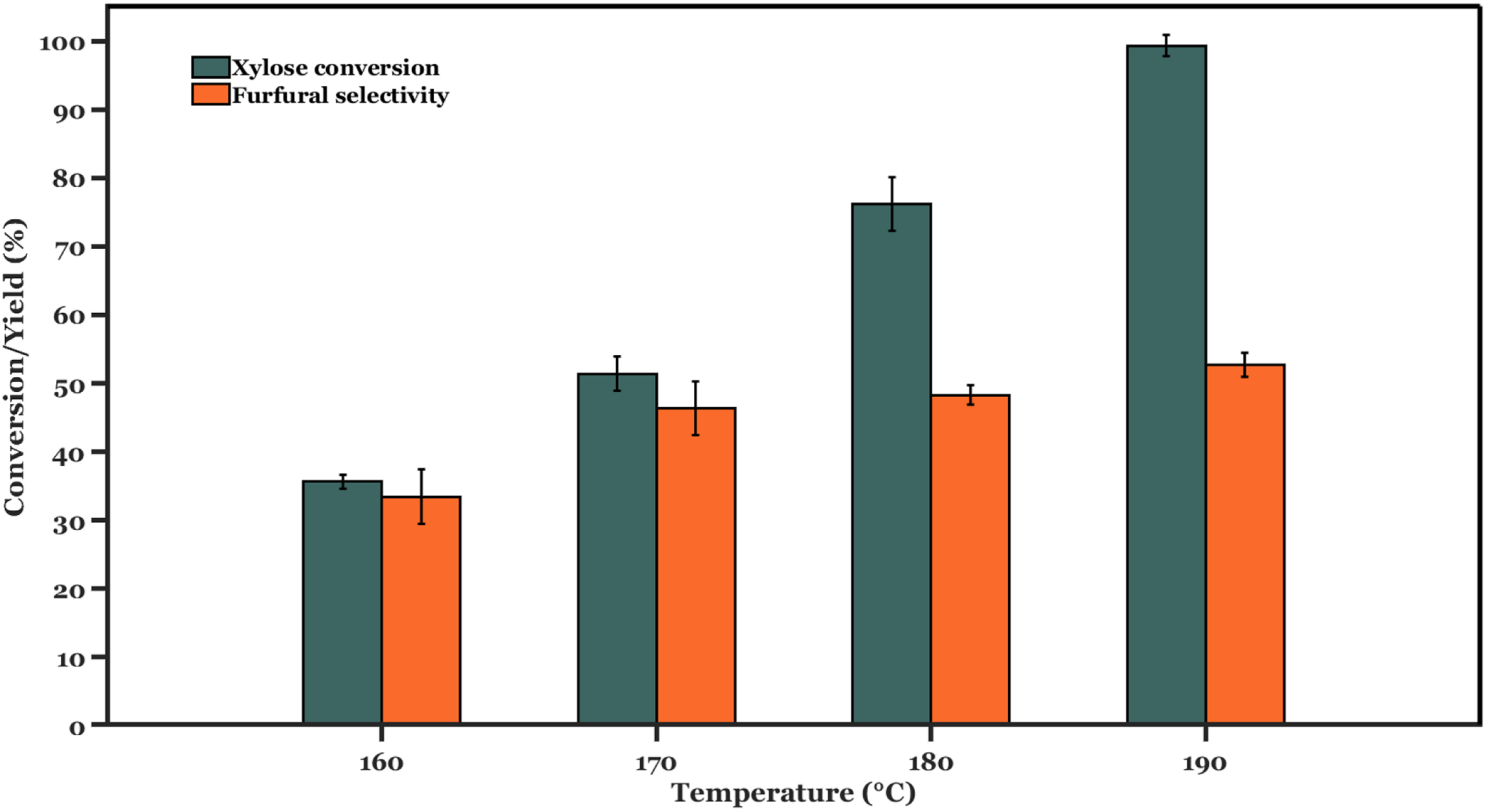
Xylose conversion and furfural selectivity using biorefinery feed over TiO_2_-coated foam catalysts. Reaction conditions: Temperature = 160–190 °C; *W*_cat_ = 90 mg; Foam density = 40 PPI; $C_{X_{0},aq}=5.7\mathrm{wt}\%$ ; *Q*_SBP_:*Q*_aq_ = 2:1 (vol.:vol.); *Q*_aq_ = 0.2 mL min^–1^.

Other works employing either homogeneous or heterogeneous catalyst systems for biphasic furfural synthesis report similar observations in terms of furfural yields with reaction temperature. Papaioannou et al.^15^ observed greater furfural yields at higher reaction temperatures while using 0.1 M H_2_SO_4_. In addition, Guo et al.^18^ reported a 10% increase in the maximum furfural yield while increasing the reaction temperature from 160 to 180 °C. Similarly, Krzelj et al.^34^ found similar furfural selectivities (55–65%) within a range of 160–190 °C using sulfonated foams. However, operating at temperatures greater than 170 °C led to deactivation due to humin formation. Li et al.^35^ also observed similar furfural selectivities (50–55%) over a wide temperature range (120–180 °C) while using a modified Ta_2_O_5_ catalyst. Increasing the temperature to 200 and 220 °C led to severe loss of furfural selectivity, assigned to furfural degradation pathways that became relevant at these extreme temperatures. According to the above, we conducted further tests at 180 and 190 °C for optimal performance.

#### 3.2.2 Effect of Solvent

While SBP is the main solvent of choice in this study, toluene was also used to perform similar experiments at 160–180 °C (see Figure S10). Our results show similar xylose conversion and furfural selectivity using both solvents as the extracting medium. These results confirm that the organic solvent does not have any major effects on sugar conversion. Or, if it had, no major differences exist between the two selected solvents. In contrast to xylose conversion, the vastly different furfural partition coefficients in toluene/water and SBP/water mixtures (i.e., ∼5–6 vs 45 $m_{\mathrm{aq}}^{3}m_{\mathrm{org}}^{-3}$ at 20 °C, respectively) were expected to play a more significant role in furfural selectivity. However, Figure S22 indicates that *m*_SBP_ decreases to <10 at reaction temperatures, whereas *m*_toluene_ has a milder decrease to 3–4 ^36^ in the same conditions. Besides, the mutual solubility of SBP increases with temperature,^37^ while in the case of toluene, it is quite low.^38^ Thus, the benefits of SBP over toluene in selectivity are, in fact, limited.

Despite the negligible effects of solvent on selectivity, a striking difference between the experiments conducted with SBP and toluene was the operability of the reactor system. In the case of toluene, an increase in pressure drop across the reactor from 0.5 to ∼32 bar in a span of 90–120 min was observed while operating at temperatures ≥ 170 °C. On the other hand, using SBP as the extractant led to a maximum increase of 2 bar in pressure drop across the reactor during ∼8 h of operation. Glucose present in the hydrolysate is converted to HMF in the presence of TiO_2_. The inability of toluene to coextract furfural and HMF (see Figure S21) results in accelerated formation of humins and their deposition on the catalyst and process lines, increasing pressure drop over operating time. This hypothesis is bolstered by relatively higher HMF yields observed while using SBP as the solvent. HMF yields increase from 9% at 160 °C to 16 and 22% while operating at 170 and 180 °C, respectively. These yields are consistently 6–7% greater than toluene as the solvent. Furthermore, our results indicate that HMF concentration in the aqueous phase while using toluene as the solvent is at least 7 times greater than in the case of SBP, due to inefficient extraction. This was corroborated by additional HMF extraction experiments (stirred batch vials), at room temperature, showing that SBP readily (co)­extracts HMF and furfural (see Figures S23 and S24). Therefore, greater aqueous HMF concentration can lead to accelerated humin formation resulting in the observed blocking of process lines while performing the experimental runs while using toluene. Other works have also shown that toluene selectively partitions out furfural rather than HMF (*m*_furfural_/*m*_HMF_ > 50).^36^ On the other hand, *m*_furfural_/*m*_HMF_ of ∼3.5 ^36^ in the case of SBP minimizes the deposition of solids and allows for continuous operation of the reactor. Thus, the effect of solvent choice plays a significant role in continuous furfural production from biomass hydrolysate and is therefore discussed in more detail in Section S3.3.2.

#### 3.2.3 Effect of Residence Time

Further, we investigate the influence of residence time at 190 °C, as shown in Figure 4. Xylose conversion increases with increasing residence time (or reduced weight-hourly space velocity, WHSV), reaching nearly full conversion at around 2.6 min. Extending the residence time also increases furfural selectivity up to around 50% at 1.7 min, while going beyond this time does not yield further improvements. Such observations point at a parallel degradation pathway that restricts furfural selectivity. To further establish if the observed selectivity trends can be explained by poor extraction efficiency, we conducted additional experiments where 3 wt % furfural was directly fed to the aqueous stream along with SBP at 170–190 °C and 3 min of residence time. Our results (Figure S15) show minimal furfural degradation at such residence times, demonstrating sufficiently fast extraction rates under these conditions. Hence, the fact that furfural selectivity remains well below 100% even at longer residence times can be attributed to the parallel formation of byproducts occurring during the conversion of xylose to furfural, thereby limiting its selectivity.

**Figure 4:**
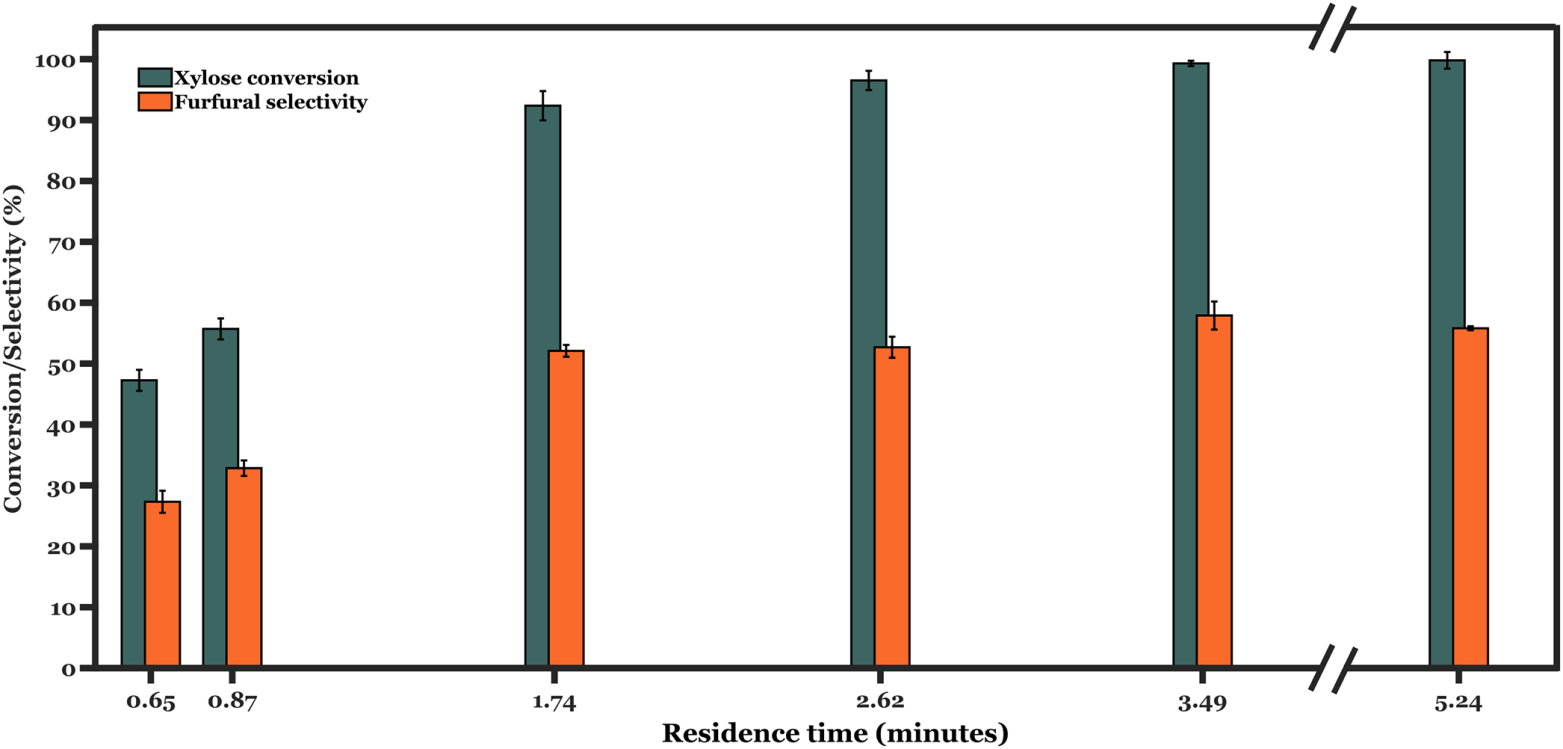
Xylose conversion and furfural stability using biorefinery feed over TiO_2_-coated foam catalysts. Reaction conditions: temperature = 190 °C; *W*_cat_ = 90 mg; foam density = 40 PPI; $C_{X_{0},aq}=5.7\mathrm{wt}\%$ ; *Q*_SBP_:*Q*_aq_ = 2:1 (vol.:vol.); *Q*_aq_ = 0.1–0.8 mL min^–1^.

In contrast to furfural selectivity, HMF yield increased from 10% at the lowest residence time (0.65 min) to 18% at 1.74 min. Further extending residence times to 5.24 min led to 34 ± 1.3% HMF yield from C6 sugars present in the feed. It should be noted that HMF selectivity could not be estimated due to the overlapping of C6 sugar peaks via the analytical method in our work. Regardless, an increase in HMF yields with residence times might indicate slower glucose conversion as compared to xylose. As shown in Figure 4, almost complete xylose conversion occurs at 1.74 min of residence time. Our experiments with model xylose and glucose feed (see Figure 6) show that approximately 50% glucose and 90% xylose are converted in the same reaction time at 180 °C. Recent works corroborate these findings of sluggish glucose dehydration.^22,39,40^

#### 3.2.4 Effect of Xylose Concentration

Several works report that lower xylose concentrations (1–2 wt %) in the feed may lead to higher furfural selectivity.^41–43^ Hence, we investigated the effect of reduced xylose concentration in the hydrolysate feed on furfural selectivity. Figure 5A shows nearly identical xylose conversion and furfural selectivity at the two feed flow rates for 190 °C with varying xylose concentration (2 and 5.7 wt %). These observations are in line with a first-order dependence on xylose concentration for its conversion. Previous works have reported similar invariant furfural selectivity over differing xylose concentrations while employing biphasic reactor operation.^15,34^ Similarly, HMF yields remain constant (within ± 2% variation) with differing feed concentrations. Furfural selectivity remains still well below 60% for all experiments conducted thus far. Therefore, maximizing furfural selectivity by varying residence times and xylose feed concentration with a certain catalyst loading remains a challenge.

**Figure 5:**
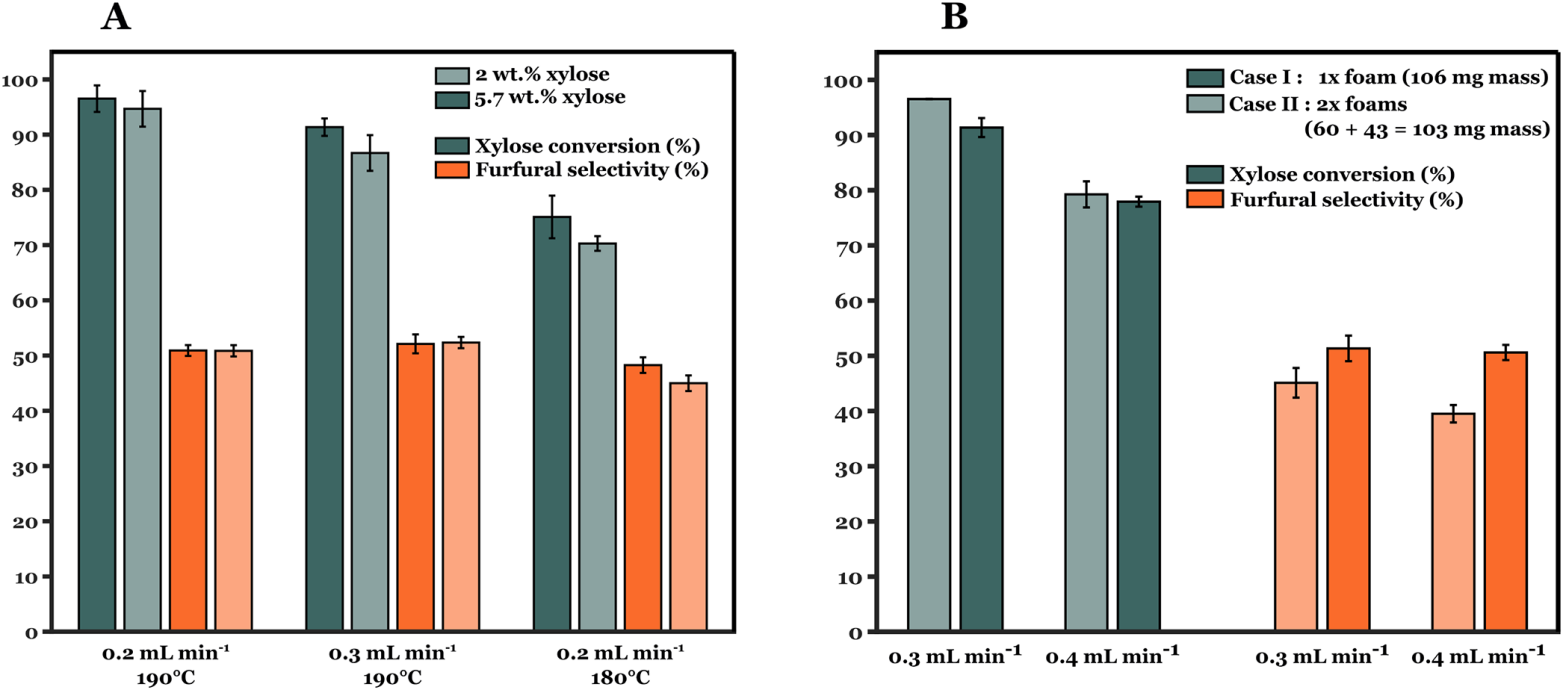
Xylose conversion and furfural selectivity using biorefinery feed over TiO_2_-coated foam catalysts. (A) Reaction conditions: *W*_cat_ = 90 mg; *Q*_SBP_:*Q*_aq_ = 2:1 (vol.:vol.) foam density = 40 PPI. (B) Reaction conditions: temperature = 190 °C; foam density = 40 PPI; $C_{X_{0},aq}=5.7\mathrm{wt}\%$ ; *Q*_SBP_:*Q*_aq_ = 2:1.

#### 3.2.5 Effect of Foam Coating Thickness

In this section, we investigated the presence of mass transfer limitations, specifically internal diffusion transport. Hence, experiments were performed with varying foam coating thickness while keeping the total catalyst mass loadings. These are presented as: case I (single foam coated with 106 mg TiO_2_) and case II (two foams coated with 60 and 43 mg TiO_2_). Case II exhibits twice the external surface area (denoted by *a*) as case I, available for external mass transfer. The washcoating thickness for case II is approximately half of that of case I based on the theoretical calculations. Therefore, case II should present shorter diffusion path lengths for both the reactants and products. Figure 5B shows the xylose conversion and furfural selectivity for both cases at different flow rates for 190 °C. Xylose conversion is slightly higher for thinner washcoat (case II). In contrast, furfural selectivity and HMF yield are better in the case of a thicker washcoat (case I—represented by the darker shade). Previous experiments show that feed dilution has no significant effect on furfural selectivity. However, a thicker coating can result in a diluted xylose concentration over the catalytic coating and also show slightly better furfural selectivities. The opposing trends observed present a complex tradeoff in choosing the optimal coating thickness.

#### 3.2.6 Effect of Feed Composition

Benchmark experiments using model xylose feed in concentrations similar to those of the biorefinery stream were performed in the flow reactor setup. Figure S16 shows the xylose conversion and furfural yields observed in both model and real feeds. There is a substantial increase in both xylose conversion and furfural yields while converting the biorefinery hydrolysate. Therefore, we systematically investigate the effects of each individual component in the biorefinery hydrolysate during xylose dehydration in a batch autoclave reactor.

Figure 6 shows xylose conversion and furfural yields in the presence of glucose, acetic acid, and galacturonic acid at 180 °C, respectively. Notably, there is no substantial difference in the trends of xylose conversion and furfural yields in the case of the model xylose solution and the respective additives. The conversion of glucose to HMF in the mixed feed with xylose is lower than that of its C5 counterpart. Other studies have shown similar behavior in the presence of solid catalysts.^22,40^ Due to the relatively low organic acid content (∼0.1 wt %) and high p*K*_a_ values (4.8 and 3.5 for acetic acid and galacturonic acid, respectively), no significant increase in the protic concentration of the aqueous media is observed. Therefore, xylose conversion is mainly affected by the presence of the solid catalyst. The presence of organic compounds in the biorefinery hydrolysate does not lead to a significant enhancement of either xylose conversion or furfural yields.

**Figure 6:**
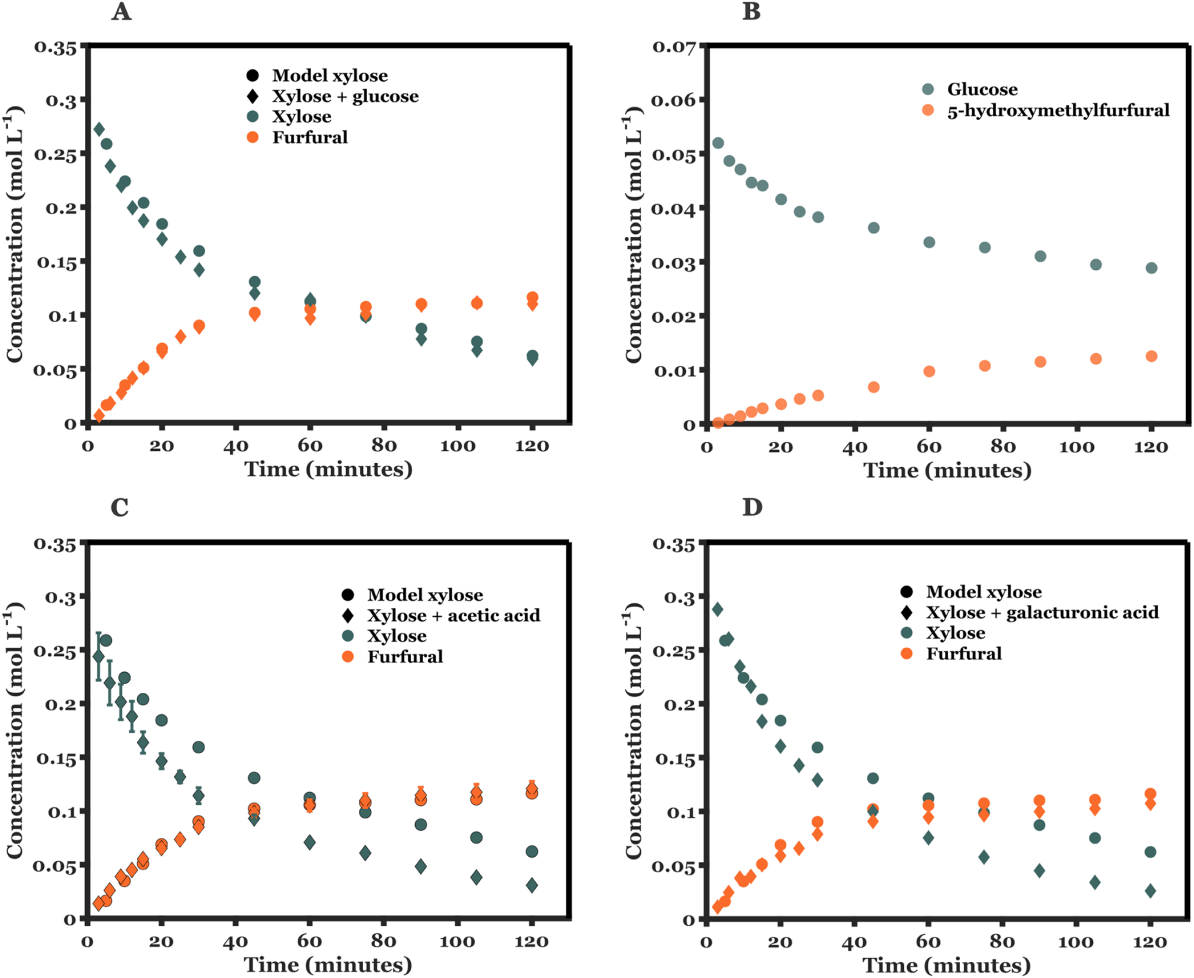
Control experiments to understand the effect of different components present in the hydrolysate on xylose dehydration at 180 °C. Concentration profiles vs time for (A) model xylose and xylose + glucose feed, (B) glucose and 5-hydroxymethylfurfural (HMF) with the mixed xylose and glucose feed, (C) model xylose and xylose + acetic acid; (D) model xylose and xylose + galacturonic acid under monophasic conditions. Reaction conditions: *T* = 180 °C; xylose: catalyst ratio = 10:1 (mass basis); $C_{X_{0},aq}=5\mathrm{wt}\%$ ; Additional components: (A) = 1 wt % glucose, (C) = 0.1 wt % acetic acid, and (D) = 0.1 wt % galacturonic acid. NOTE: Acetic acid experiments shown in (C) are performed in duplicate.

To determine the cause behind the enhanced activity using the biorefinery hydrolysate, we performed an elemental analysis of the feed using ICP-OES (description in the Supporting Information). Our results showed the presence of ∼0.5 wt% sulfur content. Although the hydrolysate was neutralized after sugar extraction from the biomass, residual sulfur still remains. To determine the content of sulfur in the form of active ${{\mathrm{SO}}_{4}}^{2-}$ present in the feed, we used ion chromatography (description in the Supporting Information). It was found that approximately 33–34 mM ${{\mathrm{SO}}_{4}}^{2-}$ remained in the hydrolysate, pointing at the presence of a small amount of (catalytically active) sulfuric acid. This translates to ∼67% of the total sulfur content detected using ICP, while the remaining 33% can be attributed to hydrolyzed feedstock proteins,^44^ which can be regarded as catalytically inactive. To prevent corrosion of the stainless steel (SS 316L) autoclave, supporting experiments with model xylose feed in the presence of 33 mM H_2_SO_4_ were performed in the flow reactor at similar conditions.

Figure 7 shows the xylose conversion and furfural selectivity obtained with three different cases listed as: case I (model xylose); case II (model xylose in the presence of 33 mM H_2_SO_4_ solution); and case III (biorefinery hydrolysate). Case I exhibits the lowest xylose conversion and furfural selectivity among the three scenarios investigated. Cases II and III, both containing ∼33–34 mM H_2_SO_4_, show superior performance than case I. Hence, it can be inferred that xylose conversion is significantly cocatalyzed by the presence of H_2_SO_4_ in the hydrolysate. The presence of the organic components such as glucose, acetic avid, and galacturonic acid (Figure 6A,C,D, respectively) does not affect the catalyst activity. Few works suggest that the presence of sulfonated groups (induced by pretreatment of bare TiO_2_, ZrO_2_, and SnO_2_ with 0.2–0.5 M H_2_SO_4_ solutions) can boost xylose conversion to furfural.^25,45,46^ Our experiments with pretreated TiO_2_ in the presence of HCl showed no significant effect in enhancing catalytic activity (see Figure S19). Therefore, it can be inferred that xylose conversion is cocatalyzed by the homogeneous H_2_SO_4_ and heterogeneous TiO_2_ counterparts. In light of these findings, the contribution of 33 mM H_2_SO_4_ toward xylose conversion and furfural formation was calculated for 180 and 190 °C (see panels B and C of Figures S11 and S12) using kinetic parameters from the work of Papaioannou et al.,^47^ for a plug flow reactor configuration. The model predictions show approximately 30% contribution of the observed catalytic activity arising from the presence of 33 mM H_2_SO_4_ in the hydrolysate for the reaction conditions used in Figure 7. In addition, few works report furfural formation from glucose and other C6 sugars.^48,49^

**Figure 7:**
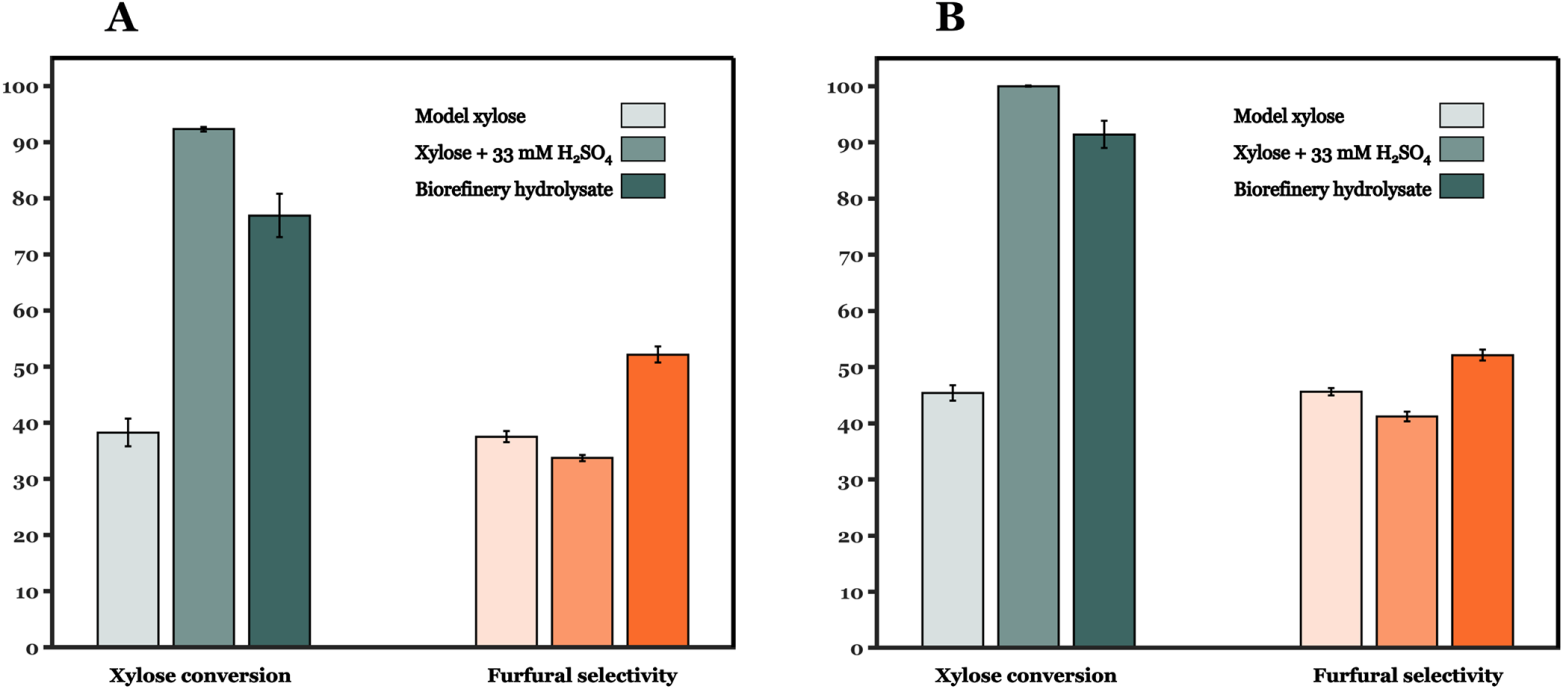
Comparison of catalytic performance of TiO_2_-coated foams while using different feeds. Reaction conditions: temperature = (A) 180 °C and (B) 190 °C; *W*_cat_ = 90 mg; foam density = 40 PPI; *Q*_SBP_:*Q*_aq_ = 2:1 (vol:vol).; *Q*_aq_ = (A) 0.2 and (B) 0.3 mL min^–1^.

#### 3.2.7 Long-Term Catalyst Stability

Previous observations show that a residence time of 2.6 min (i.e., *Q*_aq_ = 0.2 mL min^–1^ in this reactor) at 190 °C with SBP as the extractant maximizes furfural yield with a stable reactor operation. In addition, a thicker catalyst coating results in slightly better furfural selectivity with similar xylose conversion at 190 °C. For the long-term activity testing, 40 PPI foam coated with 114 mg TiO_2_ was used as the packing. Figure 8 shows relatively stable furfural selectivity (∼65–70%) over a period of 36 h of reactor operation. In the initial 2–3 h of operation, 100% xylose conversion was observed. A gradual decrease in xylose conversion occurred during 5–10 h of operation with unchanged furfural selectivity. Prolonging reactor operation after 10 h led to stable activity with respect to both xylose conversion (∼85–87%) and furfural selectivity. Approximately 38 ± 1% HMF yields are obtained during the stability testing. In addition, furfural yields obtained in this experiment is similar to other tests in this work, when residence time was normalized per mass of catalyst and per feed volumetric flow rate (mg_cat_ min mL^–1^), see Figure S14. Visual inspection of the spent foam catalyst shows a dark brown color on the coating. Calcining the spent foam at 450 °C according to its synthesis procedure led to restoration of its original coated color, indicating the formation and deposition of humins during reactor operation. This may well explain the decrease in xylose conversion in the period of 5–10 h, with several active sites being deactivated by adsorption of humic species. The coatings’ mechanical stability was verified for flow rates up to 1 mL min^–1^ (as depicted in Table S3), exceeding the reaction conditions (maximum of 0.6 mL min^–1^). Chemical inertness of TiO_2_ coatings was verified by ICP-OES analysis of the collected aqueous stream after reaction. No Ti species were detected in the aqueous media with a detection limit of 0.07 to 1 ppm.

**Figure 8:**
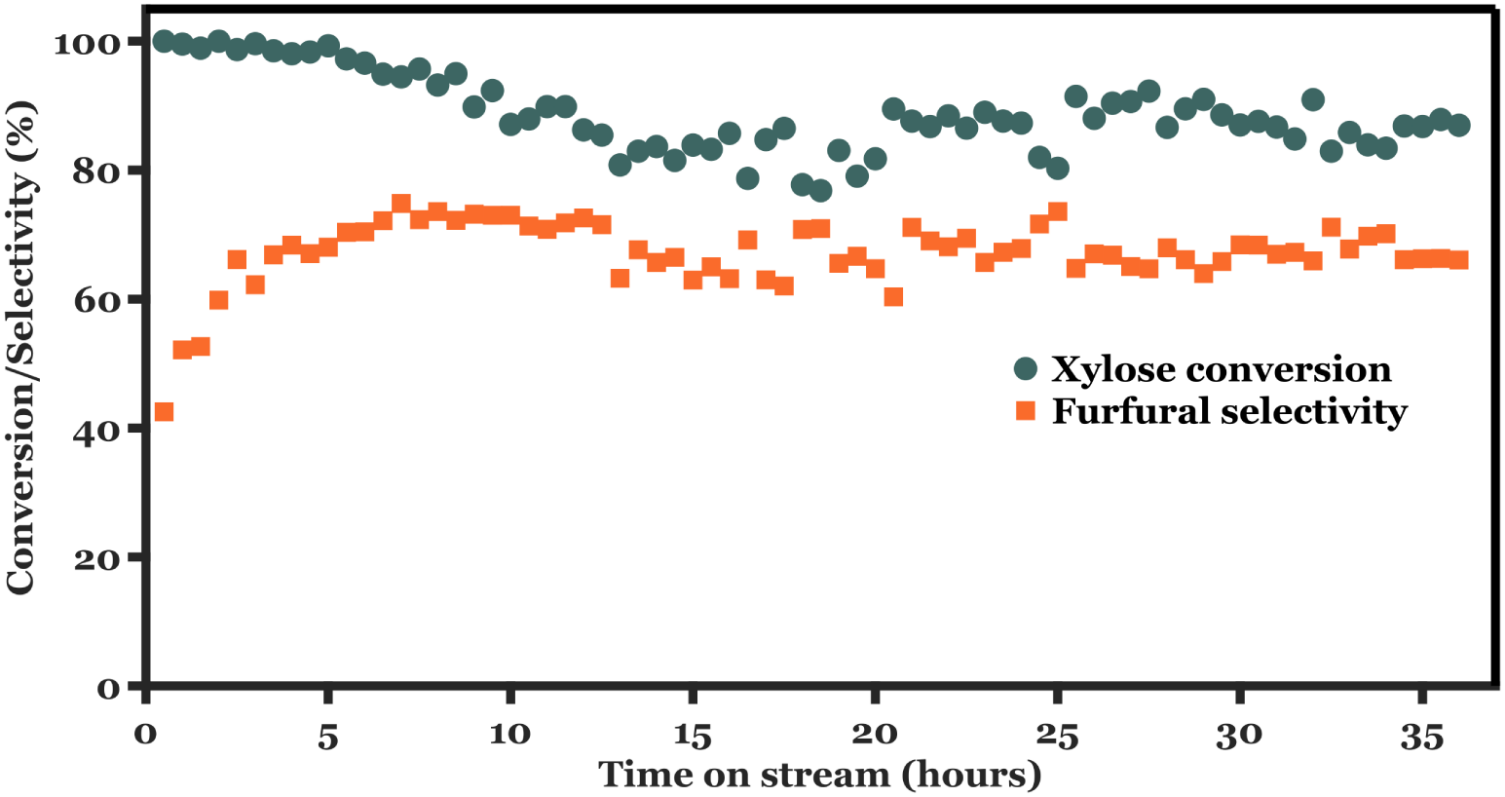
Xylose conversion and furfural selectivity using biorefinery feed over TiO_2_-coated foam catalysts. Reaction conditions: temperature = 190 °C; *W*_*cat*_ = 114 mg; foam density = 40 PPI; *Q*_aq_ = 0.2 mL min^–1^; *Q*_SBP_ = 0.4 mL min^–1^; $C_{X_{0},aq}=5.7\mathrm{wt}\%$ .

### 3.3 Practical Considerations

#### 3.3.1 Reactor Consideration

Table 3 depicts space-time-yield (STY) of furfural production in a continuous flow reactor using a heterogeneous catalyst system. Most of the studies listed use xylose as feed. Our work has used a xylose-rich hydrolysate representing a biorefinery stream. Besides, the use of coated foam structure due to its excellent mass transport properties enables reactor operation at higher temperatures (190 °C) as compared with other works. This led to a very high furfural productivity (5.85 × 10^–2^ $g_{\mathrm{furfural}}g_{\mathrm{cat}}^{-1}\min^{-1}$ ), at least an order of magnitude greater than the highest reported in the literature. Moreover, we have used a 2:1 solvent-to-aqueous ratio, which is the lowest among the other reported works (except for the previous works from our group).^15,34^ This has a significant bearing on the furfural production process (discussed in Section S3.3.2).

**Table 3:** Literature Data for Furfural Yields Obtained with Different Xylose Concentrations and Organic Solvent Ratios Using Continuous Reactor Configuration

Catalyst	Solvent	Solvent ratio $\left(m_{\mathrm{org}.}^{3}m_{\mathrm{aq}.}^{-3}\right)$	Xylose (wt %)	Furfural yield (%)	STY $\left(g_{\mathrm{furfural}}g_{\mathrm{cat}}^{-1}\min^{-1}\right)$	. Temp. (°C)	E-factor	s-STY^a^	Reference
Amberlyst-36 +	MIBK	9:1	5	70	9.15 × 10^–4^	130	1.23	1.27 × 10^–4^	^26^
Ga-USY (1:2)	MIBK	4:1	5	40	1.04 × 10^–3^	130	2.90	3.24 × 10^–4^	^26^
Ta_2_O_5_-p	1-butanol	1.5:1	10	59	1.6 × 10^–3^	180	1.64	1.32 × 10^–3^	^35^
0.1 M H_2_SO_4_	Toluene	2:1	4	54	-	190	1.89	-	^15^
HND-580	CO_2_	3:1	5	21–22	1.00 × 10^–3^	170	6.10	5.38 × 10^–3^	^43^
${{\mathrm{SO}}_{4}}^{2-}/\gamma -{\mathrm{Al}}_{2}O_{3}$	CO_2_	3:1	5	18	1.04 × 10^–3^	170	7.68	5.59 × 10^–3^	^43^
${{\mathrm{SO}}_{4}}^{2-}/\gamma -{\mathrm{Al}}_{2}O_{3}$ + HND-580	CO_2_	3:1	5	34	2.08 × 10^–3^	170	3.59	1.12 × 10^–2^	^43^
Sulfonated foams	Toluene	2:1	6	40	-	200	2.90	-	^34^
β-zeolite	GVL^b^	8.6:1^c^	2	70	1.79 × 10^–3^	160	1.23	1.98 × 10^–4^	^50^
TiO_2_-coated foams	SBP	2:1	5.7	57	4.62 × 10^–2^	190	1.74	2.36 × 10^–2^	This work
TiO_2_-coated foams	SBP	2:1	5.7	31	5.02 × 10^–2^	180^d^	4.04	2.57 × 10^–2^	This work
TiO_2_-coated foams	SBP	2:1	5.7	57	5.85 × 10^–2^	190^e^	1.74	2.99 × 10^–2^	This work

a s-STY is expressed as furfural productivity per mass of solvent used $\left(g_{\mathrm{furfural}}g_{\mathrm{cat}}^{-1}g_{\mathrm{solvent}}^{-1}{\mathrm{hr}}^{-1}\right)$ .

b Monophasic cosolvent system.

c 9:1 (weight:weight basis).

d 0.4 mL min^–1^.

e 0.3 mL min^–1^.

E-factor is an important sustainability metric depicting the amount of waste generated per furfural. Hence, values close to 0 indicate a more atomically efficient process. E-factor values for this work are comparable with other continuous furfural production reports using biphasic solvent system due to similar yields, ranging from 50 to 70%. Similarly, another sustainability metric capturing furfural productivity per mass of hazardous substance used, i.e., mass productivity, was found to be comparable for all the works listed in Table 3. However, this parameter does not capture the varying amount of catalysts used in different reports. Therefore, we normalized the furfural productivity per mass of solvent used in each process to obtain s-STY (solvent-incorporated space-time yield), expressed as $g_{\mathrm{furfural}}g_{\mathrm{cat}}^{-1}g_{\mathrm{solvent}}^{-1}{\mathrm{hr}}^{-1}$ . The benefits of using low catalyst loadings at a high reaction temperature (190 °C) due to the foam structure’s excellent mass transport properties allow for a 2–3-fold increase of s-STY, showcasing superior sustainability metrics per mass of furfural produced.

Several studies have consistently indicated that the activation energy for this reaction typically falls within the range of 111–125 kJ mol^–1^,^41,51,52^ especially under acidic conditions. In our investigation, we similarly discovered that the activation energy for this reaction in a flow reactor is approximately 91.6 kJ mol^–1^ (determined in a small temperature range of 160–180 °C, as shown in Figure 9), which aligns closely with the value obtained from batch reactor experiments, around 86 kJ mol^–1^. Notably, Lu et al.^43^ and Aellig et al.^26^ reported much lower activation energies, around 54 kJ mol^–1^. Both works ascribe the reduced activation energy to reactor type. However, the intrinsic kinetics for a given catalyst should remain unaffected by reactor configuration. On the other hand, it is well established that, for heterogeneous catalyzed reactions, reaction kinetics can be falsified if determined under external and/or internal mass transfer limitations,^53,54^ resulting in activation energies of ∼2–5 kJ mol^–1^ or half of its true value, respectively. The latter case may be true in both reported works, as the smallest particle sizes used (i.e., 200 and 500 μm) are substantially larger than the washcoat thickness (15–20 μm) used in this work, potentially leading to severe diffusion limitations. In our case, using 90–110 mg TiO_2_ coatings (20 μm thickness) at 190 °C leads to a Weisz–Prater modulus of ∼0.12. Based on these calculations, no internal diffusion limitations exist for an ∼35 μm TiO_2_ coating. In addition, the diffusion time scale (0.6–1 s) is significantly faster than the reaction time (210 s), confirming reaction-limited kinetics. For biphasic operation with open-cell foams, external mass transfer time scales (liquid–solid for xylose, solid–liquid and liquid–liquid for furfural extraction) should be smaller than the reaction time to maximize furfural extraction over its degradation. These time scales are significantly shorter in the case of catalyst-coated foams (see Section S6 for a detailed explanation). Similarly, time scales of diffusion, mass transfer and reaction should be considered during reactor design.

**Figure 9:**
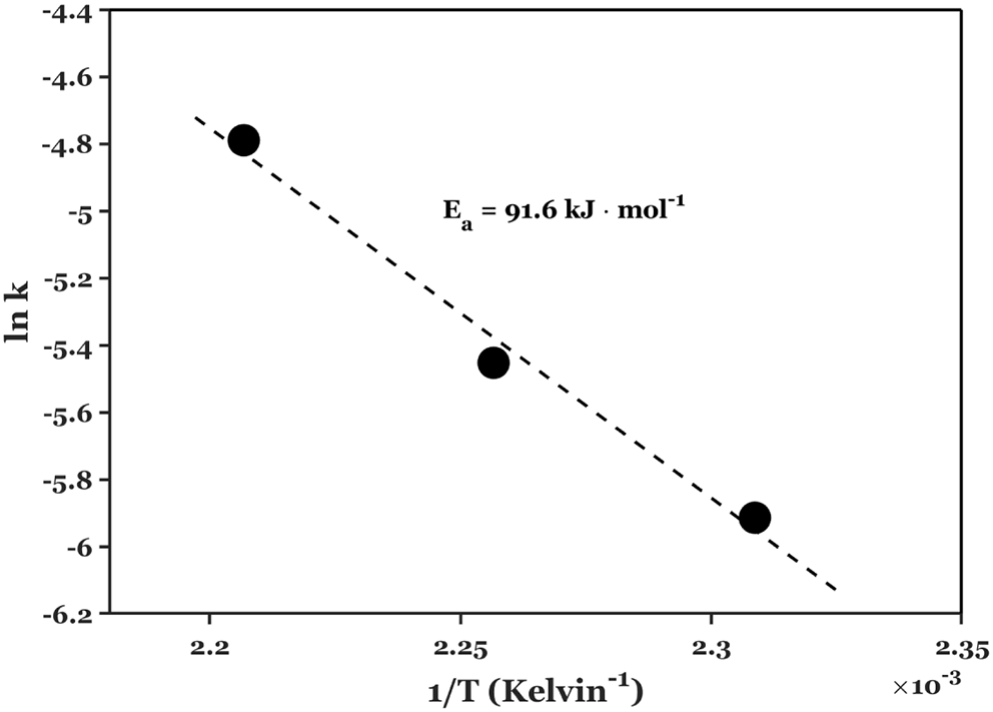
Activation energy for initial xylose dehydration using biorefinery feed over TiO_2_-coated foam catalysts. Reaction conditions: temperature = 160–180 °C, *W*_cat_ = 90 mg, foam density = 40 PPI, *Q*_aq_ = 0.2 mL min^–1^, *Q*_SBP_ = 0.4 mL min^–1^, $C_{X_{0},aq}=5.7\mathrm{wt}\%$ .

Following the potential effects of mass transfer limitations in this reaction, high liquid–liquid mass transfer rates due to Taylor flow regime have been claimed while using micropacked bed reactors (μ-PBRs).^55–57^ However, this advantage is at the expense of high pressure drop when using catalytic particle sizes <100 μm. Despite their tortuous structure of interconnected aluminum ligaments, coated open foam structures do not result in high pressure drops when used as a packing due to their open-cell structure (bed voidage > 0.85 for 40 PPI foams). This construction induces turbulence which results in high mass transfer rates, albeit reducing the catalyst mass loading per unit of reactor volume. Increasing the catalyst loading by increasing the washcoat thickness (>35 μm for xylose dehydration at 190 °C or higher reaction temperature) will inadvertently lead to an increase in the characteristic length for diffusion of reacting molecules, likely leading to significant internal diffusion limitations and reducing the overall catalytic efficiency.

#### 3.3.2 Other Process Considerations

The employment of a biphasic system, coupled with a heterogeneous catalyst for xylose dehydration, introduces its own set of advantages in downstream processing. Additionally, despite variations in the C5 sugar concentrations in the hydrolysate obtained from biomass pretreatment, TiO_2_-coated open-cell foams can be operated with relatively unchanged furfural selectivity using SBP as organic solvent. This provides flexibility in terms of different biomass feedstocks potentially considered for this process. By seamlessly reincorporating unused xylose, this closed-loop approach not only minimizes waste but also enhances the overall efficiency and sustainability of the biorefinery. To ensure continuous operation for converting a hemicellulose-rich hydrolysate, an organic solvent should possess three important characteristics. First and foremost, a high value of *m*_furfural_ at reaction conditions is crucial since furfural is the primary target product of the process. High *m*_furfural_ enhances furfural yield by enhancing the extraction rates (see eq 1). Similarly, high *m*_HMF_ enables the extraction of HMF (produced via C6 sugar dehydration). Not only does it yield a valuable byproduct, but it also serves to mitigate the formation of humins. HMF, if left in the reaction medium, can undergo unwanted reactions leading to the formation of humins, which can negatively impact the efficiency and continuity of the operation. Second, the solvent should not degrade during operation and be inert to furfural. Other solvents exhibiting high *m*_furfural_ and *m*_HMF_ such as 2-butanol,^58^ ethyl acetate,^59^ and MIBK^60^ have been used. However, Qi et al.^61^ discovered that MIBK forms a byproduct due to an aldol condensation reaction with furfural, resulting in the formation of C_11_H_14_O_2_. Third, the ideal solvent must be less volatile than furfural. Since furfural concentration in the organic phase cannot be greater than 3 wt %. (even assuming 100% furfural selectivity), using solvents that are more volatile than furfural will lead to extremely high energy demands and separation costs. This emphasizes the complexity of solvent selection in biphasic systems and highlights the importance of a comprehensive understanding of solvent behavior to ensure optimal process performance.

In this study, a maximum of 60–72% furfural selectivity was obtained. Although this does not significantly surpass previous studies, the STY or furfural productivity in this work is notably higher than any other reported in the literature. This is attributed to the use of more severe reaction temperatures (depicted in Table 3), which was possible because of the fast mass transfer rates induced by the 3D-coated structures. It should be noted that other works require much greater contents of organic solvent than those used in this study to achieve similar yields. Using lower organic solvent content translates into a lower furfural storage capacity, and thereby could cause lower furfural yields. On the other hand, using a greater solvent content not only increases CAPEX (i.e., larger equipment sizes) and operational costs (i.e., energy requirements for pumping and covering heating/cooling needs), but might even lead to poor catalyst wetting by the aqueous feed, rendering lower xylose conversion. Hence, solvent content effects must be thoroughly optimized and understood from a process perspective.

Besides the importance of solvent in making process choices, furfural selectivity ranging from 50 to 67%, as achieved in this work, and negligible solids formation, as evident due to continuous long-term operation (see Figure 8), indicate the formation of soluble byproducts. Analysis of the organic phase using GC-MS shows the presence of furanic species in the organic extractant phase (see Figure S21). Therefore, these byproducts predominantly exist in the aqueous phase. GC-MS analysis of the aqueous phase shows the presence of acetic acid, formic acid, lactic acid, and pyruvaldehyde, and corroborated by other works.^62^ The “contamination” of aqueous phase by the presence of these byproducts in dilute concentrations will present a challenge for recycling of the aqueous phase due to the accumulation of these inert molecules. While purging a part of this aqueous stream can prevent their accumulation, it comes at the cost of additional wastewater treatment. Alternative strategies such as aqueous phase reforming (APR) of the aqueous phase exiting the reactor can be performed to yield valuable longer-chain hydrocarbons. However, current investigations into such technologies are performed at higher concentrations of the organic molecules.^63^

Finally, suspended solids in biorefinery feeds and insoluble humins formed during this process can block filters and lines, eventually causing intermittent reactor operation. Therefore, we propose a parallel flow reactor system (see Figure 10), inspired from the filter skid used in the feed pretreatment in vacuum gas oil hydrotreating unit in a conventional oil and gas refinery. A reactor packed with coated foam structure can be used with an inline filter at its outlet. With continuous monitoring of the pressure drop across each reactor, an on-demand backwash of individual reactor can be performed when necessary. This system ensures continuous furfural synthesis from the biorefinery hydrolysate stream.

**Figure 10:**
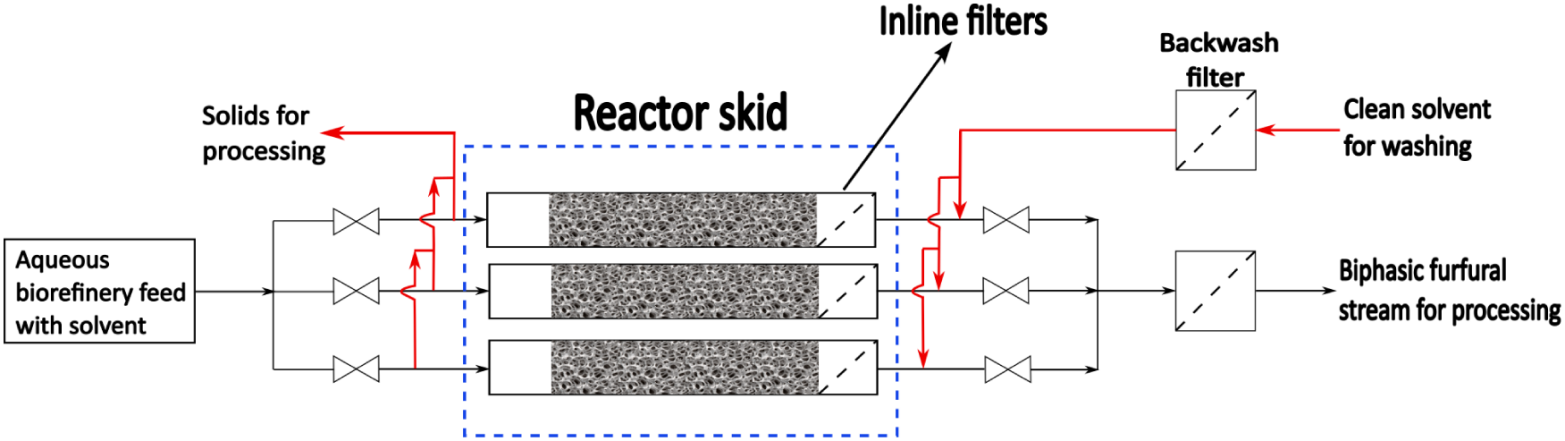
Proposed operational scheme with parallel flow reactors packed with catalytically active 3D foam structures.

## 4 Conclusions

This study employed TiO_2_-coated on open-cell foam structures as catalyst packing for biphasic furfural synthesis from a biorefinery hydrolysate, consisting predominantly of xylose with organic acids, glucose, and ∼33 mM H_2_SO_4_, in a continuous flow reactor. The geometry of the open-cell foam structures facilitated hydrodynamic mixing between the two liquid phases, enabling in situ extraction of furfural. TiO_2_ coatings were found to be stable under flow conditions. SBP and toluene as the organic solvent affect neither the catalytic activity of TiO_2_ nor furfural selectivity during biorefinery hydrolysate conversion. Nevertheless, SBP can also coextract HMF produced from glucose in the hydrolysate, which is crucial for continuous reactor operation by minimizing solids formation.

Reducing the concentration of xylose in the feed from 5.7 wt % to 2 wt % did not result in an increase of furfural selectivity. The presence of C6 sugars in the real hydrolysate stream does not hinder the C5 sugars reaction, while organic acids such as acetic acid and galacturonic acid present in negligible quantities, do not significantly affect xylose conversion and furfural selectivity. The presence of ${{\mathrm{SO}}_{4}}^{2-}$ ions in the form of H_2_SO_4_ from biomass pretreatment cocatalyzes furfural production from the prehydrolysate mixture. Regardless, high operating temperatures (>180 °C) and use of a fast mass transfer rates in 3D-coated foam structured were key to maintain promising yields and minimize residence time (<3 min), which is the lowest reported in the literature using heterogeneous catalyst. Additionally, operating under such severe conditions also enabled high furfural productivity of 5.85 × 10^–2^ g_furfural_ g_cat_ min^–1^, at least 25 times greater than the highest reported in the literature. Additionally catalyst activity of TiO_2_-coated foam structures was found to be stable for at least 36 h of operation at 190 °C.

## Supplementary Material



## Notes

Detailed experimental procedures, characterization, and relevant catalytic activity data (PDF)
