# Supplementary material for: Structured Catalysts for Continuous Biphasic Furfural Synthesis from Biorefinery Feedstock
Source: ACS Sustain Chem Eng. 2025 Oct 14;13(42):17856–68. doi: 10.1021/acssuschemeng.5c05251 (PMC12570264; doi:10.1021/acssuschemeng.5c05251)
Supplement: Supplementary file 1 [file sc5c05251_si_001.pdf]

Supplementary Information

Structured catalysts for continuous biphasic furfural synthesis from biorefinery feedstock

Adarsh Patil, Afnan Ahmad, Fernanda Neira d'Angelo*[a]

[a] Chemical Reactor Engineering Laboratory, Sustainable Process Engineering,

Eindhoven University of Technology, Eindhoven 5600 MB, The Netherlands

E-mail: m.f.neira.dangelo@tue.nl

Number of pages: 34

Number of figures: 25

Number of tables: 4

Contents

1 Literature overview **S1**

2 Methodology **S1**

2.1 Catalytic foam preparation S1

2.2 Activity testing S3

2.2.1 Flow reactor for biphasic experiments S3

2.2.2 Batch autoclave reactor for monophasic experiments S4

2.3 Analytical techniques S5

2.3.1 HPLC and GC S5

2.3.2 Parametric definitions S6

2.3.3 ICP-OES S7

2.3.4 IC S7

2.4 Powdered TiO_2 characterization S8

2.4.1 N_2 physisorption S8

2.4.2 XRD S8

2.4.3 NH_3-TPD S8

2.5 Foam characterization methods S8

2.5.1 SEM S8

2.5.2 Foam stability testing S9

3 Foam pre-treatment and coating results **S11**

4 Catalytic testing **S15**

4.1 Catalyst screening results S15

4.2 Flow experiments S16

4.3 Autoclave experiments S24

4.4 GCMS analysis of reaction samples S27

5 **Partitioning experiments with organic solvents** **S27**

6 **Mass transfer time scales** **S30**

6.1 Liquid-liquid extraction S30

6.2 Solid-liquid mass transfer S31

6.3 Furfural degradation and formation rates in 33 mM H_2SO_4 and TiO_2 . . . . S31

6.4 Calculation of kinetic parameters for TiO_2 . . . . S32

References **S33**

1 Literature overview

Table S1: Literature review for furfural synthesis from hydrolysate mixtures using acid catalysts.

Feed	Catalyst	Solvent	Temp. (°C)	Time (min.)	Yield* (%)	E-factor -	M.P.[&]	Ref.
Birch	-	SBP^B	190	180	56	1.79	0.50	[1]
Corncob	$Biochar^b$	DCM^B	170	60	83	0.88	0.83	[2]
Corn stover	$H_2SO_4{}^a$	$MIBK^B$	170	20	80	0.95	1.53	[3]
Corn stover	Purolite $CT275^b$	$MIBK^B$	170	20	71.5	1.18	1.36	[3]
Eucalyptus	$H_2SO_4{}^a$	$Water^M$	160	60	32.4	3.82	0.79	[4]
Eucalyptus	$H_2SO_4{}^a$	$Water^M$	160	60	37.6	3.22	0.91	[4]
Hardwood	Acetic $acid^d$	$MIBK^B$	170	100	80.1	0.95	0.98	[5]
Hardwood	Acetic $acid^d$	$Water^M$	170	100	37.7	3.22	0.81	[5]
Wood chips	Acetic $acid^d$	$Water^M$	190	100	28	4.58	0.61	[6]
Wood chips	$H_2SO_4{}^a$	$Water^M$	170	100	48	2.25	1.04	[6]
Pulp	$H_2SO_4{}^a$	$Water^{\#,G}$	160	120	68	1.29	2.07	[7]
Maple	$H_2SO_4{}^a$	$Water^M$	190	91	50	1.74	1.71	[8]

* - Furfural yield, & - mass productivity and E-factor as defined by Sheldon,[9] *a* - Homogeneous catalyst, *b* - Heterogeneous catalyst, *B* - Biphasic system, *M* - Monophasic system, *d* - present in the feed, *G* - Continuous reactor, # - Autogeneous steam stripping.

2 Methodology

2.1 Catalytic foam preparation

Aluminium foams were chosen as substrate due their ease of handling, low cost, good thermal conductivity. Aluminium foams (purchased from ERG Aerospace: 40 pores per inch, aka PPI, and 1120 $m^2\,m^{-3}$) were cut in cylinders (1 cm diameter and 2 cm length, weighing 106.4 mg). These cylinders are then exposed to a sequence of pre-treatment steps, as sketched in Figure S1, which are further described below to induce catalytic activity.

The aluminium foams were first cleaned in an ultrasonic bath with isopropanol at 40°C and then dried. Subsequently, these were etched with 0.1 M NaOH in an ultrasonic bath at 40°C for 10 minutes. Next, electro-oxidation of the etched foams was performed in 0.8 M oxalic

acid electrolyte. The schematic of the anodization cell can be found in Figure S1. Lead was used as the cathode. Isothermal anodization was performed at a constant current density of 2 $A\,m^{-2}$, corresponding current of 1.17 A and an initial cell voltage of 42 V, at 40°C using a heating jacket. The cell voltage increased from 42 to 58 V in 30 minutes. The aluminium foams after anodization exhibited a distinct golden hue (see Figure S5) substantiating the reproducibility of the pre-treatment. To remove traces of organics from the surface of foam, the foams underwent calcination at 550°C for 6 hours at 2°C min^{-1}.

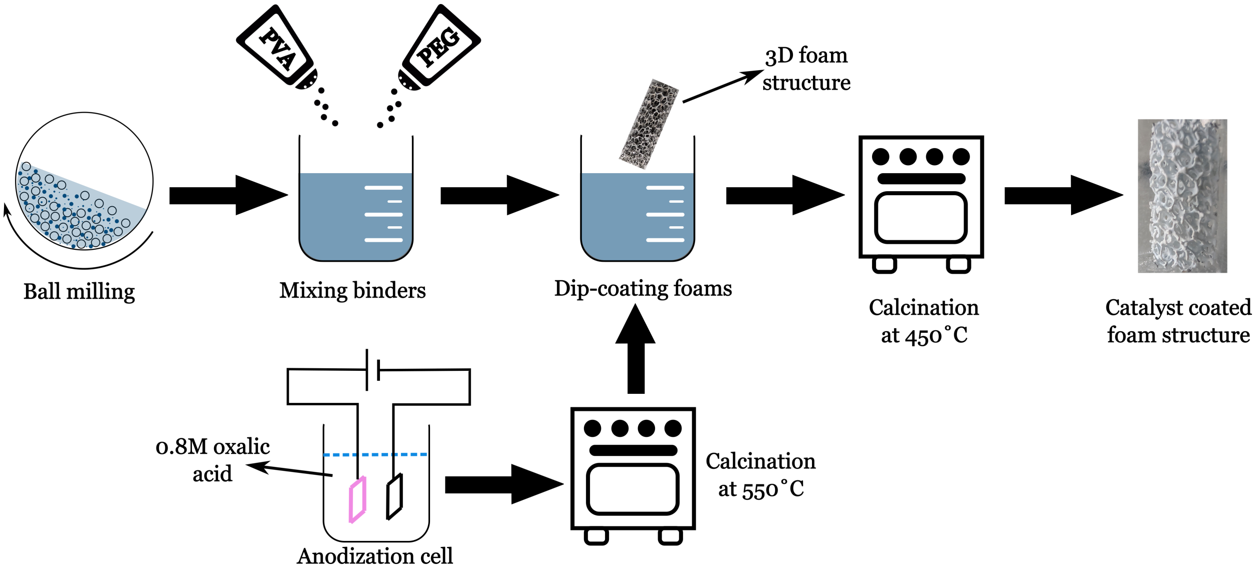

Figure S1: Synthesis procedure for catalytic foam preparation showing the different steps involved.

The washcoat suspension was prepared by mixing equal mass of solutions A and B. Solution A is a suspension of solid catalyst in water. It consists of 20 wt.% TiO_2 (Degussa P-25, procured from Sigma-Aldrich and consisting of 80-20% mix of anatase and rutile phases, respectively) in water obtained after (planetary) ball milling the required mass of TiO_2 and water for 30 minutes. Solution B consists of 6 wt.% polyvinyl alcohol (PVA) and 4 wt.% polyethylene glycol (PEG), used as binders, in water. For a typical dip-coating procedure, the calcined foam was first immersed in the prepared washcoat suspension. Pressurized air passing through an air gun was then used to remove the excess washcoat material on the foam. Initially, the pressure was kept to a minimum for removing the exterior washcoat

excess. After the appearance of a more viscous washcoat (occuring as a result of evaporation through forced convection) at the interior of the foam structure, air pressure was increased further to remove the residual recalcitrant washcoat. The coated foams were further dried for 2 hours at 60°C. Multiple layers of washcoat were achieved by replicating the aforementioned dipcoating steps including drying. Upon reaching a suitable washcoat mass, the coated foams were calcined for 4 hours at 450°C (2°C min^{-1}) where binders were decomposed. This results in a drop in the mass of washcoat. The washcoat loading (W_L) of samples was determined by the difference between the final weight of the coated foam after each dipcoat ($W_{washcoat}$) and the initial weight of the metallic foam ($W_{substrate}$).

2.2 Activity testing

2.2.1 Flow reactor for biphasic experiments

A sketch of the experimental setup used for catalyst foam testing is provided in Figure S2. In a regular experiment, 4 foam blocks are packed inside a 12.7 mm ID, 10 cm long Hastelloy (HC-22) tubular reactor equipped with silver lined-SS frits acting as filters at the reactor inlet and outlet. The catalyst loading was based on the mass uptake on the foam structures obtained after calcination. A single foam was coated for all experiments with the other foam packings being un-coated. The reactor is vertically arranged in a downflow configuration kept in a circulating air oven. An ISCO Teledyne pump and Knauer HPLC pump was used to feed the organic solvent (toluene and SBP) and the aqueous biorefinery feed (5.7 wt.% xylose content), respectively. These two liquids are pre-mixed, and the resulting biphasic mixture flows co-currently downwards through the reactor, which under reaction conditions operates at 160–200°C and 25 bar pressure. The total pressure, which is kept greater than the vapor pressure of water and the organic solvent to prevent boiling of the liquid streams, is controlled by feeding high pressure nitrogen to a 500 ml vessel connected to the reactor outlet and a back pressure regulator on the gas outlet line. The liquid outlet stream is collected in the same vessel, that also ensures pressure stability while sampling. Each experimental

condition is evaluated under steady state conditions with regular sampling at an interval of 20 minutes.

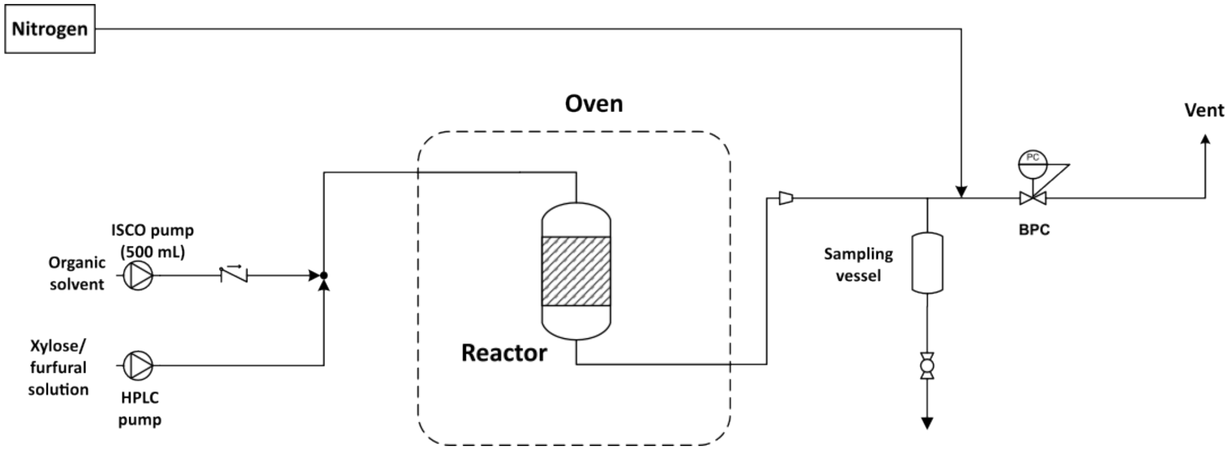

Figure S2: Scheme of reactor setup used for catalyst testing.

2.2.2 Batch autoclave reactor for monophasic experiments

Catalytic activity testing was performed in a stainless steel autoclave reactor (200 mL volume) with an external heating jacket as shown in Figure S3. For a typical experiment, 0.8 gram of catalyst was placed with approximately 120 grams of water in the autoclave. The remaining water (total required = 152 grams) along with 8 grams of xylose were then placed in an overhead injection vessel at room temperature and pressurized at a pressure greater than the water saturation pressure at reaction temperature. Initially, the catalyst and water in the jacketed autoclave were heated to the desired reaction temperature. After reaching the desired reaction temperature, the injection vessel with pressurized nitrogen and its contents were quickly emptied into the autoclave. This was recorded as time zero. Samples were obtained from the overhead sampling line immersed in an ice bath to cool the liquid sample to room temperature to prevent undesired water evaporation which may otherwise lead to falsified concentration of organic species in the sample.

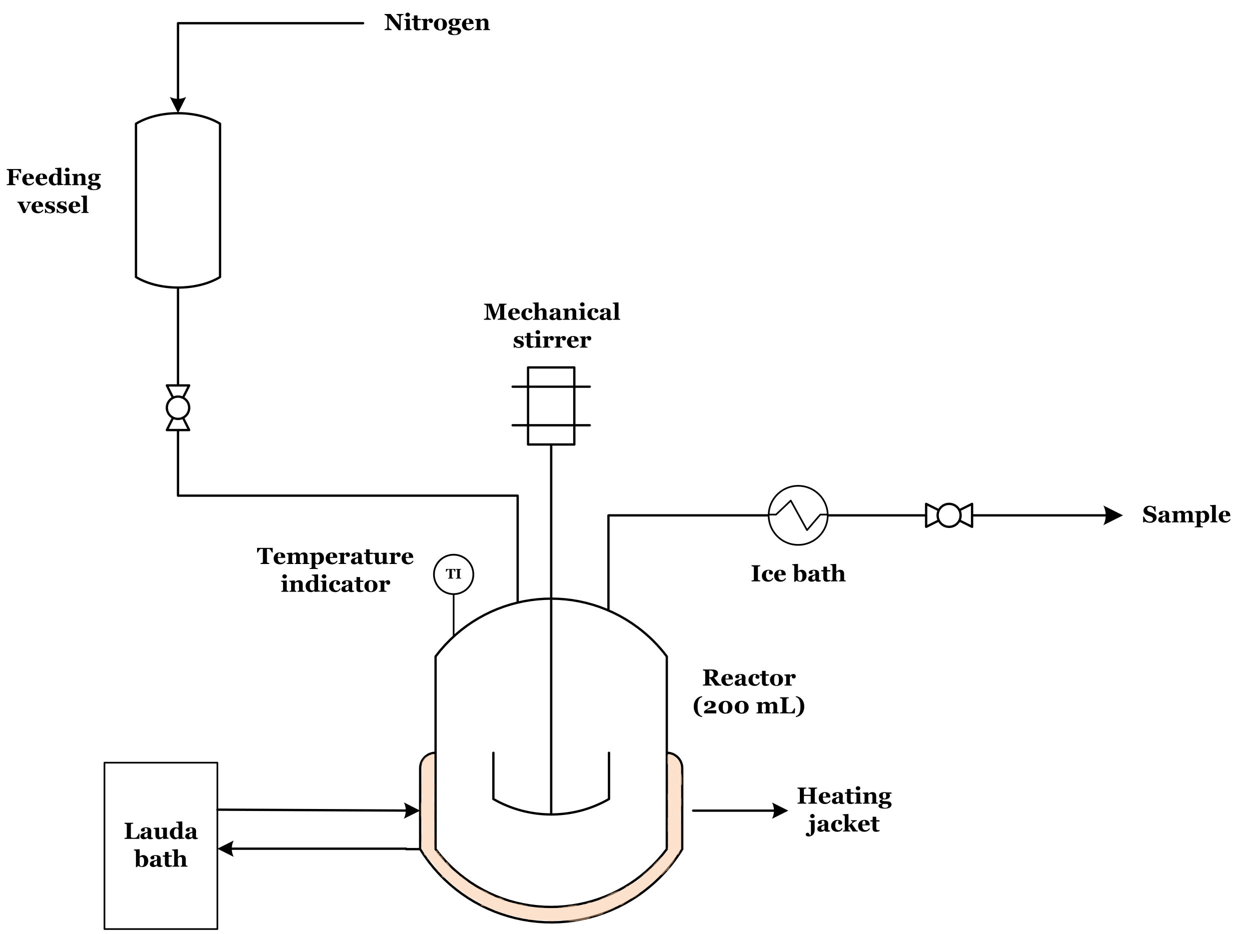

Figure S3: Schematic of the autoclave setup used for catalytic testing

2.3 Analytical techniques

2.3.1 HPLC and GC

Analysis of the liquid samples is done by a combination of HPLC and GC for the aqueous and organic phases, respectively, using the following instruments and methods: HPLC Shimadzu (SIL-20A) with an Agilent Metacarb 67C column, water as mobile phase, 70°C, RI detector (Waters 2414) for xylose determination in the aqueous phase; HPLC Shimadzu (SIL-20A) with a Zorbax Phyenyl column using methanol:water (20:80) as mobile phase, 60°C, UV-vis detector (SPD-M20A, $\lambda = 254$ nm) for furfural determination in the aqueous phase; and GC (Varian CP-3800) with a Varian capillary FactorFour CB Sil 5 CP column, He as mobile phase, linear heating from 60 to 200°C and FID detector for furfural determination in the

organic phase.

2.3.2 Parametric definitions

The following definitions of residence time (τ), xylose conversion (X_{xylose}), furfural selectivity ($S_{furfural}$) and yield ($Y_{furfural}$) were used to interpret the data for flow reactor:

$$\tau = \frac{V_R \cdot \epsilon_{aq}}{Q_{aq}} = \frac{V_R \cdot \epsilon_{org}}{Q_{org}} \tag{S1}$$

$$X_{xylose}(\%) = \frac{C^{inlet}_{aq,xylose} - C^{outlet}_{aq,xylose}}{C^{inlet}_{aq,xylose}} \cdot 100 \tag{S2}$$

$$S_{furfural}(\%) = \frac{C^{outlet}_{aq,furfural} \cdot Q_{aq} + C^{outet}_{org,furfural} \cdot Q_{org}}{(C^{inlet}_{aq,xylose} - C^{outlet}_{aq,xylose}) \cdot Q_{aq}} \cdot 100 \tag{S3}$$

$$Y_{furfural}(\%) = \frac{C^{outlet}_{aq,furfural} \cdot Q_{aq} + C^{outlet}_{org,furfural} \cdot Q_{org}}{C^{inlet}_{aq,xylose} \cdot Q_{aq}} \cdot 100 \tag{S4}$$

where $C_{phase,i}$ is the concentration of the species i in the designated phase (aq and org stand for aqueous and organic phase, respectively), V_R is the reactor volume excluding the volume of the solid packing, and ϵ_{phase} is the volumetric holdup of each phase. The latter is defined as the ratio between the volumetric flow of that phase (i.e., Q_{phase}) and the total liquid flow rate assuming complete immiscibility (i.e., Q_{aq} and Q_{org}). Although water is a product of the dehydration reactions, due to the low feed concentrations, the total volumetric flow rates are assumed constant throughout the reaction. It should also be noted that furfural was not present in the feed of the reactor for all the experiments conducted in this work.

Similarly, the following definitions of xylose conversion, furfural yield and selectivity were used to interpret the data for batch experiments under monophasic conditions:

$$X_{xylose}(\%) = \frac{C^{t=0}_{xylose} - C^{t}_{xylose}}{C^{t=0}_{xylose}} \cdot 100 \tag{S5}$$

$$S_{furfural}(\%) = \frac{C^{t}_{furfural}}{C^{t=0}_{xylose} - C^{t}_{xylose}} \cdot 100 \tag{S6}$$

$$Y_{furfural}(\%) = \frac{C^{t}_{furfural}}{C^{t=0}_{xylose}} \cdot 100 \tag{S7}$$

2.3.3 ICP-OES

Inductively Coupled Plasma - Optical Emission Spectroscopy (iCAP^{TM} PRO, Thermo Scientific^{TM}) was used to analyze the content of S in the aqueous biorefinery feed. For this, standard solution of differing H_2SO_4 concentrations were prepared, followed by the analysis of the biorefinery feed (filtration done using 0.2 μm PVDF filter).

Since the reactor was packed with aluminium foams and the SS frits on either side of the packing being silver-lined, we checked Ag and Al content of the aqueous phase after processing to check leaching in presence of H_2SO_4. Ag was not observed (detection limit = 100 ppb), while only 1 ppm of Al was detected in the aqueous phase.

2.3.4 IC

Dionex^{TM} Integrion^{TM} HPIC chromatography system (Dionex, Sunnyvale, CA, USA), consisting of an isocratic pump, an anion pre-column (Dionex Ionpac, AG11-HC-4 μm RFIC, 2 $\times$ 50 mm), an anion separator column (Dionex Ionpac, AG11-HC-4 μm RFIC, 2 $\times$ 250 mm), coupled with an anion self-regenerating suppressor (Dionex CDRS 600), a Dionex integrator and a conductivity detector was used to analyze ${SO_4}^{2-}$ ions present in the biorefinery feed. 30 mM KOH, used as an eluent, was generated in situ using a Dionex EGC 500 KOH RFIC and HPIC eluent generator cartridge, connected to a continuously regenerated trap column (CR-TC, Dionex). An isocratic flow rate of 0.38 mL min^{-1} was used. Standard solutions prepared for ICP-OES analysis were used for IC analysis as well to obtain a calibration curve.

2.4 Powdered TiO_2 characterization

2.4.1 N_2 physisorption

Next, the textural analysis of the specimens was conducted by low temperature (88 K) N_2-physisorption using Micromeritics TriStar II 3020.The specific surface area was determined by Brunauer-Emmet-Teller (BET) equation,[10] while the total pore volume (V_{tot}) was obtained at the relative pressure $P/P_0 = 0.99$ using BJH method.[11]

2.4.2 XRD

X-ray powder diffraction (XRD) patterns were obtained by a Rigaku MiniFlex 600 diffractometer with Cu-Kα (0.154 nm) source and equipped with a Kβ (x2) filter with constant step of 0.02 °2θ and counting time of 2 seconds per step. Intensity at angles between 10-90°was measured at a rate of 0.6 °min^{-1}.

2.4.3 NH_3-TPD

NH_3-temperature programmed desorption (TPD) measurements were performed in AutoChem II 2920 equipped with a thermal conductivity detector. For a typical measurement, the powdered samples were first degassed at 200°C with He, followed by NH_3 adsorption (2% in He) at 90°C. He was flowed over the catalytic bed to remove physisorbed NH_3 followed by temperature ramp of 2°C min^{-1} from 100 to 500°C in presence of He.

2.5 Foam characterization methods

2.5.1 SEM

The washcoat thickness of the foams were analyzed using scanning electron microscopy (SEM - SU8020, Hitachi, Tokyo, Japan). For this purpose, several parts of the foam after coating were cut using a pair of scissors to clearly distinguish between the coating and foam

substrate. The observed value can be compared with the theoretical thickness (d) according to washcoat mass ($W_{washcoat}$), foam volume ($V_{substrate}$), porosity (ϵ_w) that was determined as 0.52 for TiO_2, (determined using BET), particle density (δ_p), based on the bulk support material density and lastly, the pore volume (V_{pore}) obtained by the BET method.

$$\mathrm{d} = \frac{V_{washcoat}}{S_{geo}\, V_{subs}} = \frac{W_{washcoat}}{\delta_p\,(1-\epsilon_w)\, S_{geo}\, V_{subs}} \tag{S8}$$

$$\delta_p = \frac{1}{\frac{1}{\delta_{\mathrm{TiO2}}} + V_{pore}} \tag{S9}$$

2.5.2 Foam stability testing

A flow test using a syringe pump to flow the reaction solvent (i.e., water) with a rate of 1 $\mathrm{mL\,min^{-1}}$ with the coated foam used as a packing in a quartz tube reactor (as shown in Figure S4).

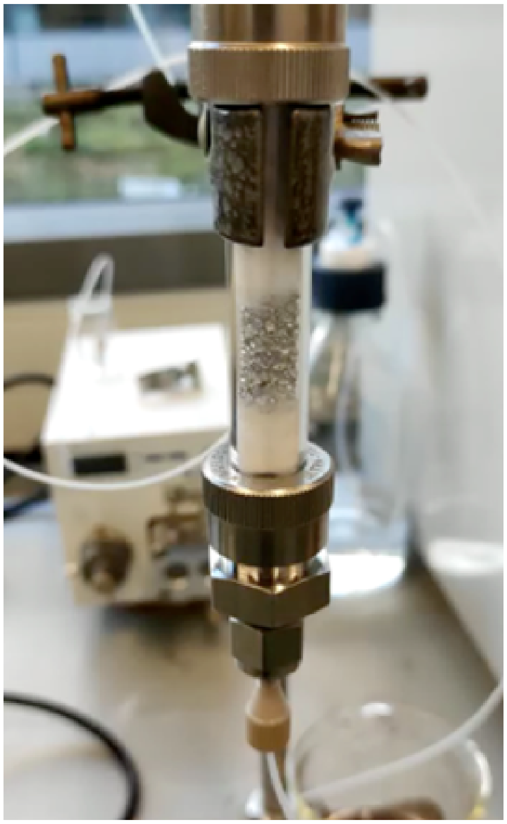

Figure S4: Quartz glass reactor with coated foams as packings to investigate mechanical stability of coatings.

3 Foam pre-treatment and coating results

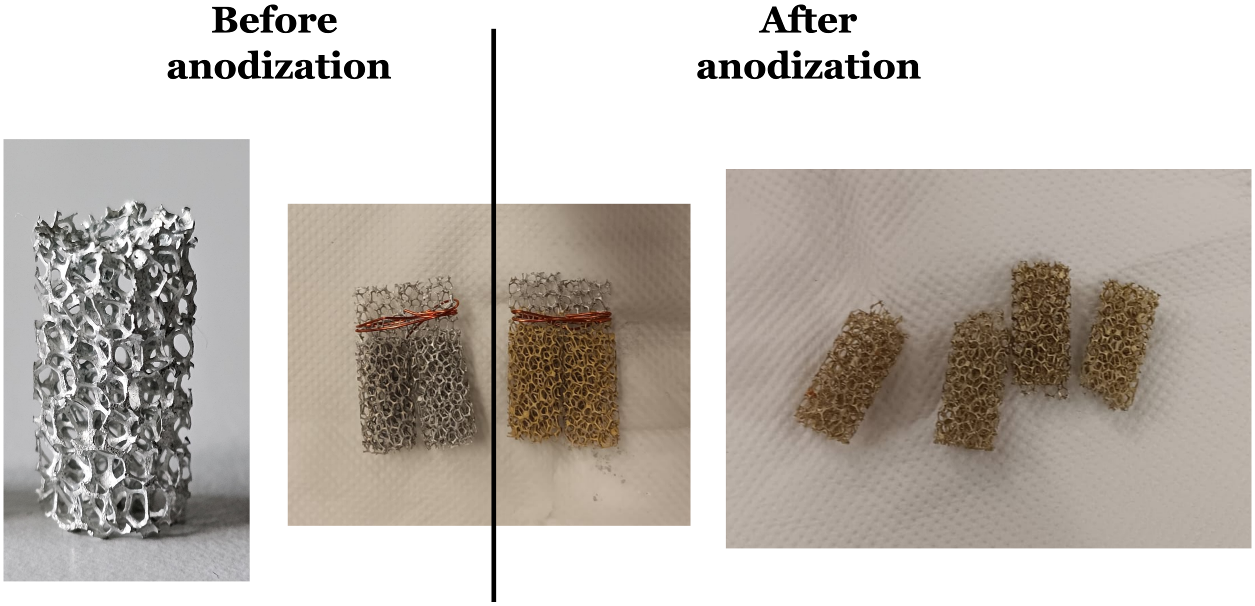

Figure S5: 3D open cell aluminium foams before and after anodization. A distinct golden hue can be seen after anodization pre-treatment.

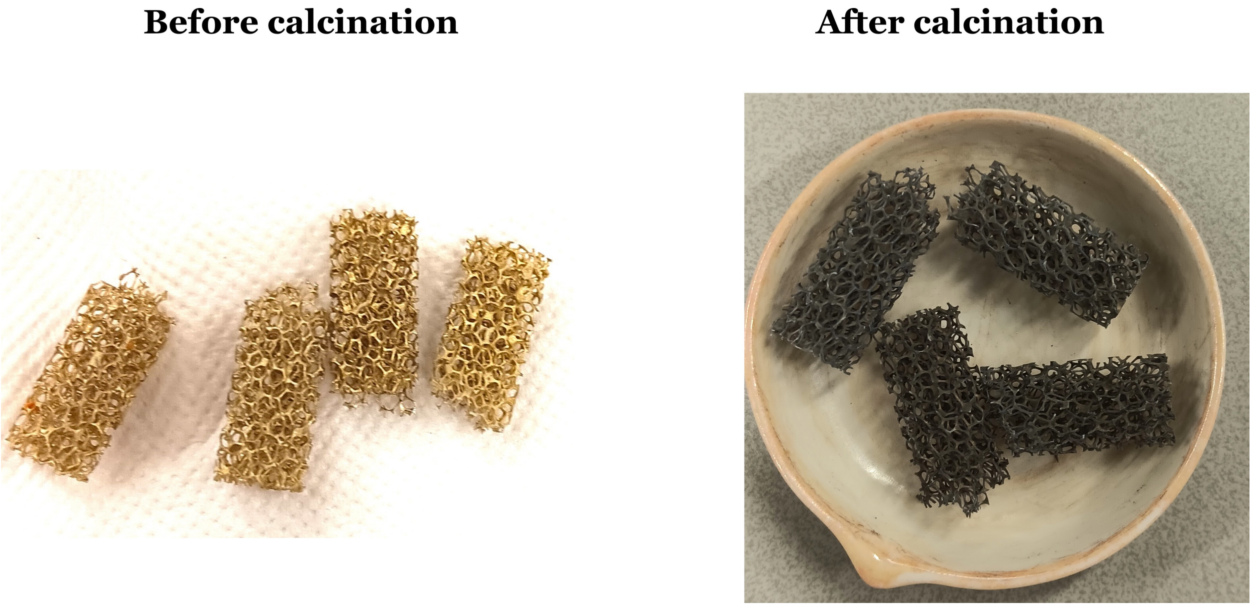

Figure S6: 3D open cell aluminium foams before and after calcination at 450°C.

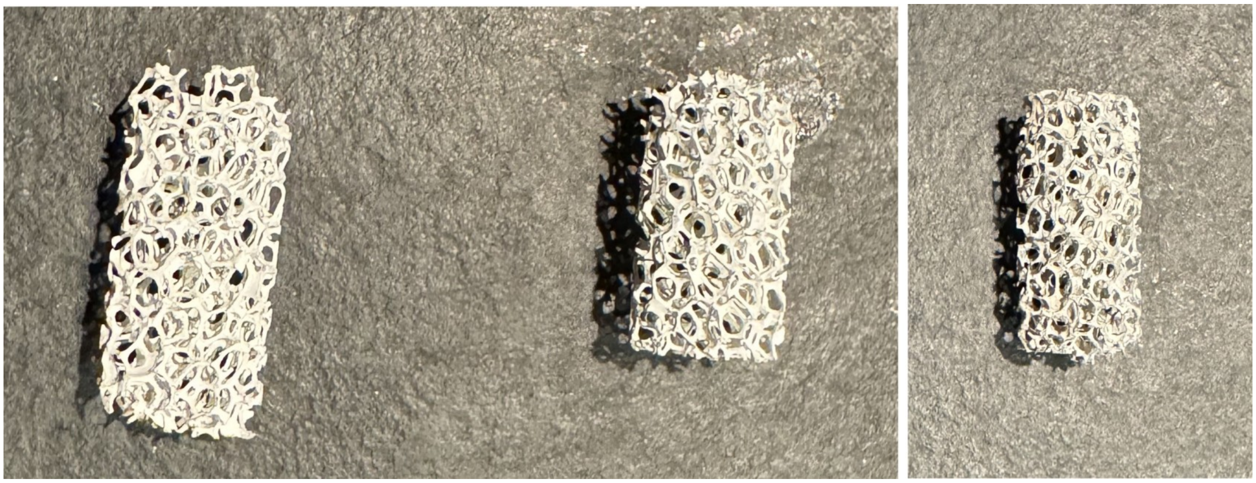

Figure S7: 3D open cell aluminium foams after dipcoating with TiO_2 and calcination at 450°C.

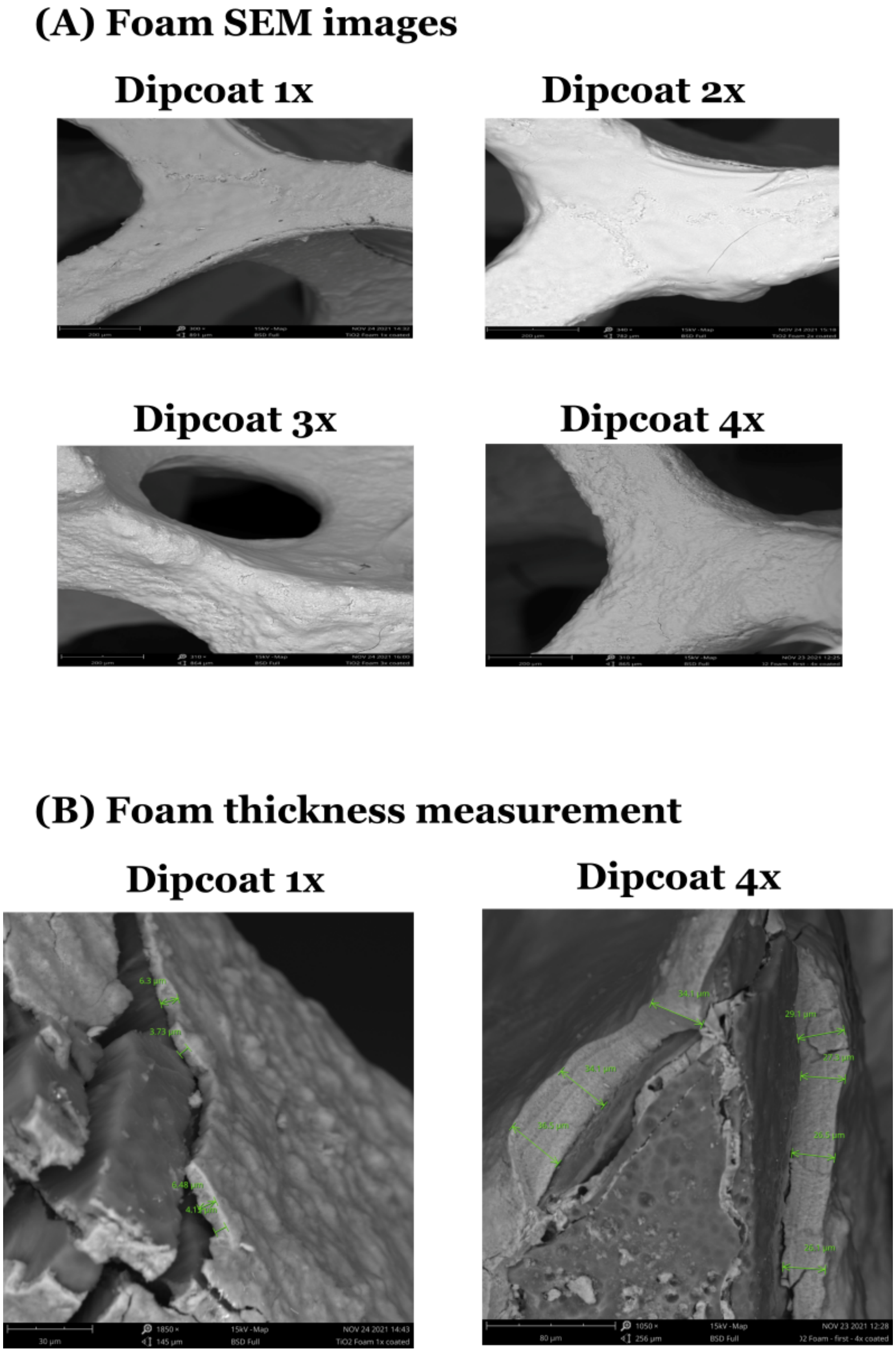

Figure S8: (A): SEM images of foams after coating; (B): SEM images used for thickness measurement of the wash-coat

Table S2: Mass uptake of aluminium foams (pore density = 40 PPI) after dip-coating and calcination treatment in a 10 wt.% TiO_2 and 2+3 wt.% (PVA+PEG) in water slurry.

Entry	No. of dip-coats	Mass uptake before calcination mg	Mass uptake after calcination mg	Weight loss of coating after calcination %
1	5	143.8	100	30.4
2	4	117.1	79.7	31.9
3	4	131.2	90	31.5
4	4	116.2	76	34.5
5	4	111.1	81.8	26.3

Table S3: Foam stability testing results

Foam weight before stability test (grams)	Foam weight after stability test (grams)	Mass loss (milligrams)
0.373	0.3722	0.8
0.310	0.309	1
0.357	0.356	1

4 Catalytic testing

4.1 Catalyst screening results

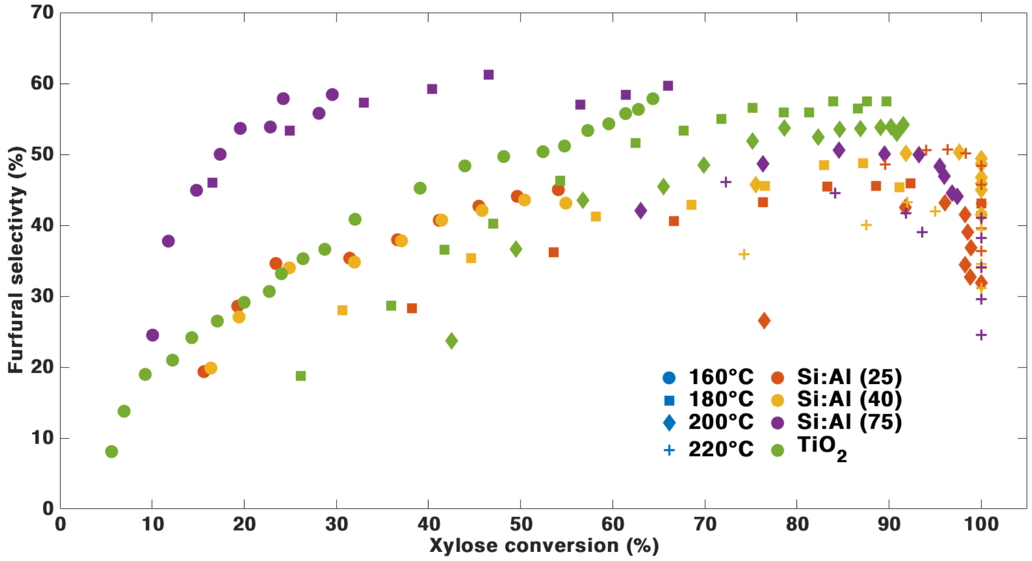

Figure S9: Furfural selectivity *vs.* xylose conversion for different catalyst screened using autoclave reactor. Reaction conditions: T = 160-200°C; W_{cat} = 0.8 grams; W_{xylose} = 8 grams; C_{xylose} = 5 wt.% in water; Stirrer speed = 600 RPM.

4.2 Flow experiments

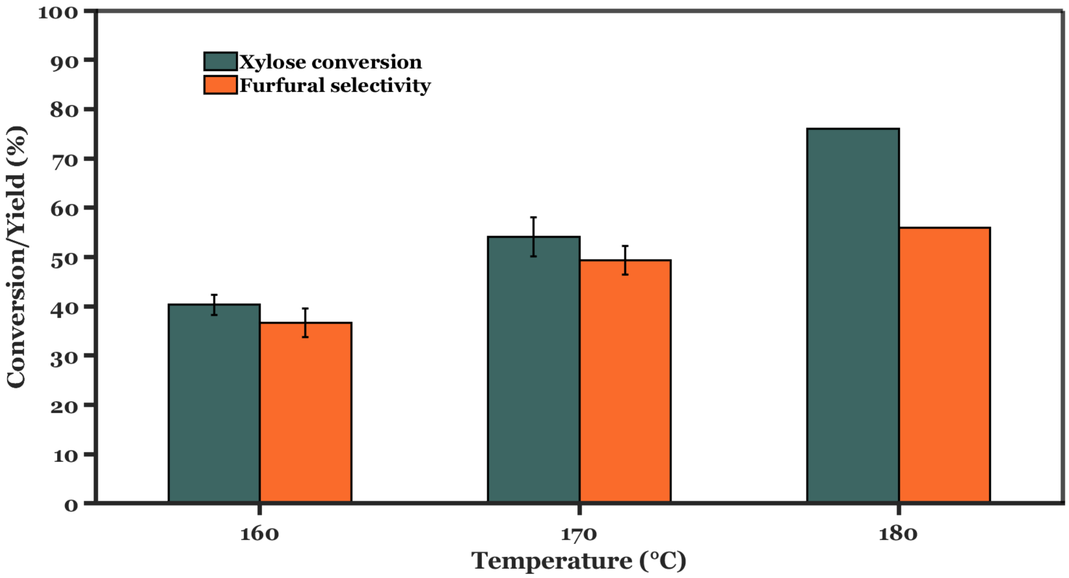

Figure S10: Xylose conversion and furfural selectivity using biorefinery feed over TiO_2 coated foam catalysts. Reaction conditions : Temperature = 160-180°C; W_{cat} = 90 mg; Foam density = 40 PPI; $C_{X_0,aq}$ = 5.7 wt.%; Q_{aq} = 0.2 mL min^{-1}; $Q_{toluene}$:Q_{aq} = 2:1 (vol.:vol.). **NOTE**: Datas with error bars represent experiment performed in duplos.

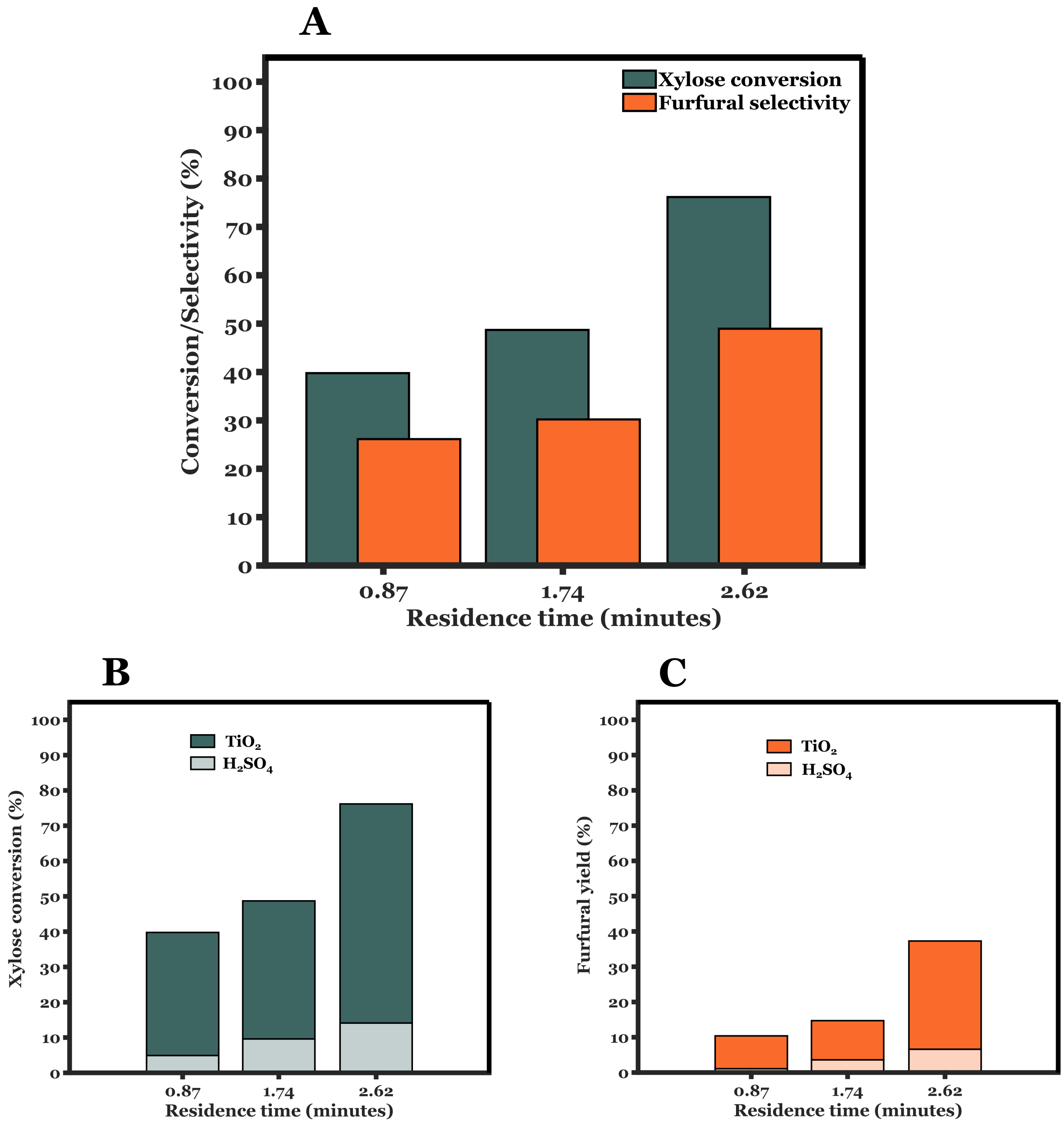

Figure S11: Xylose conversion and furfural selectivity using biorefinery feed over TiO_2 coated foam catalysts. Reaction conditions : Temperature = 180°C; W_{cat} = 90 mg; Foam density = 40 PPI; $C_{X_0,aq}$ = 5.7 wt.%; $Q_{toluene}$:Q_{aq} = 2:1 (vol.:vol.); Q_{aq} = 0.2-0.4 mL min^{-1}.

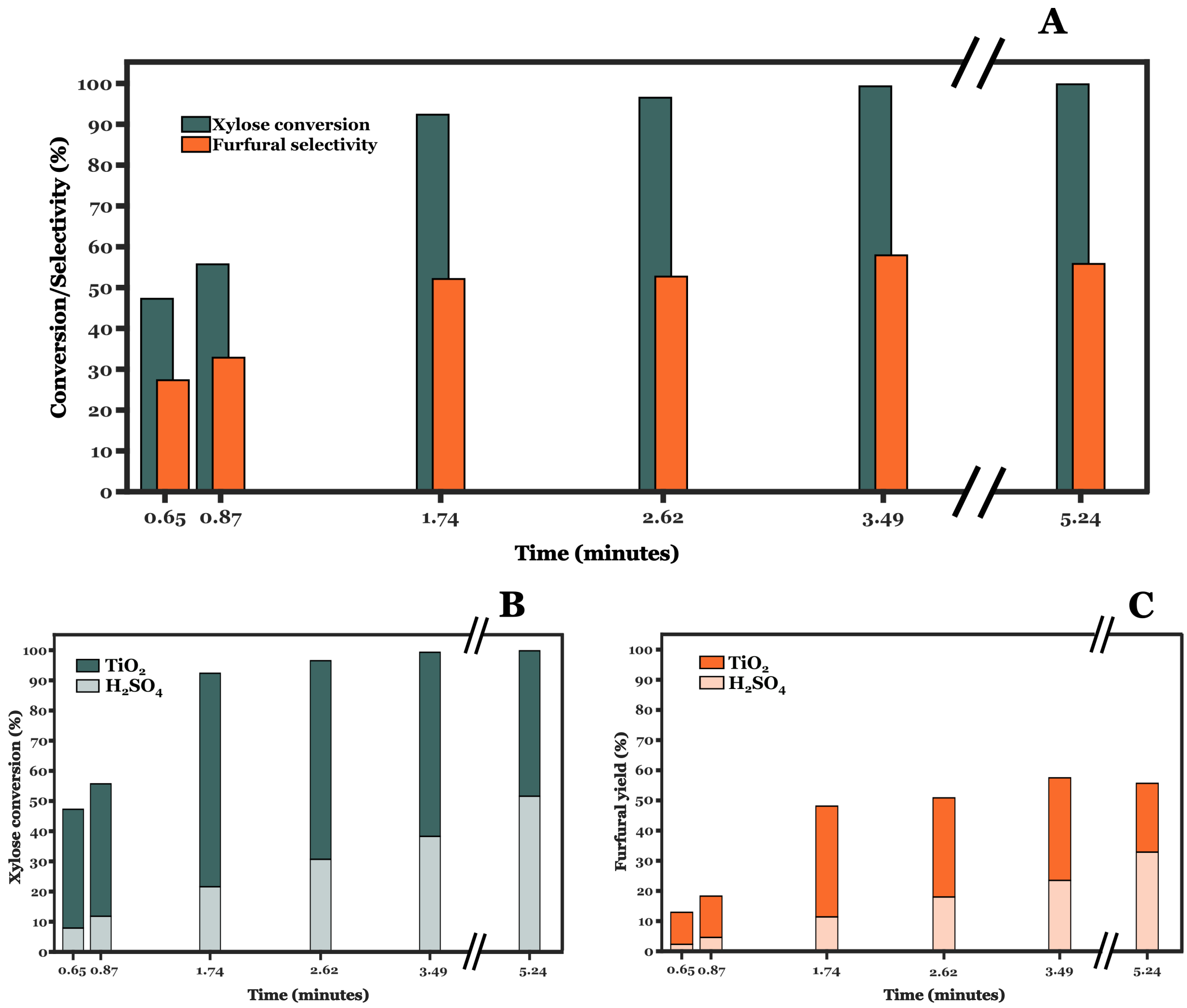

Figure S12: Xylose conversion and furfural selectivity using biorefinery feed over TiO_2 coated foam catalysts. Reaction conditions : Temperature = 190°C; W_{cat} = 90 mg; Foam density = 40 PPI; $C_{X_0,aq}$ = 5.7 wt.%; $Q_{toluene}$:Q_{aq} = 2:1 (vol.:vol.); Q_{aq} = 0.1-0.8 mL min^{-1}.

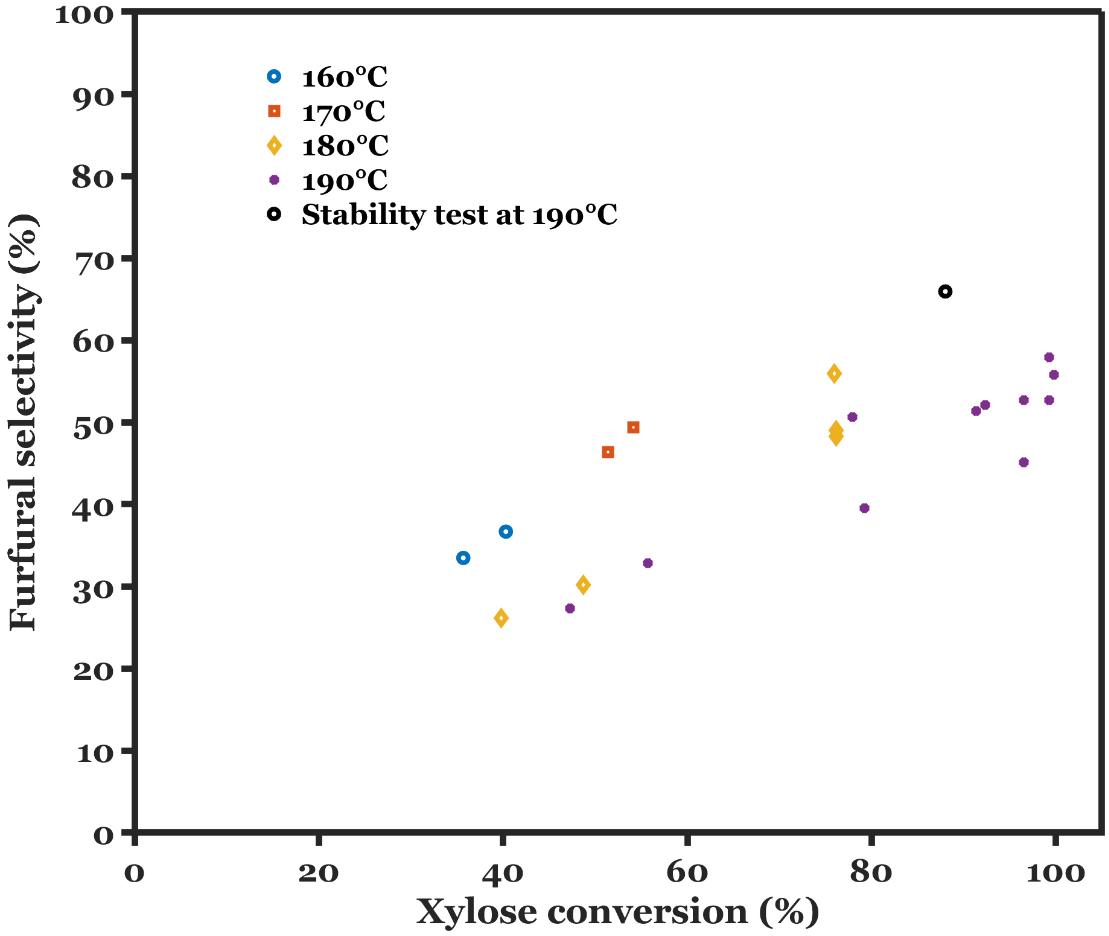

Figure S13: Furfural selectivity as a function of xylose conversion for all the experiments performed in this work.

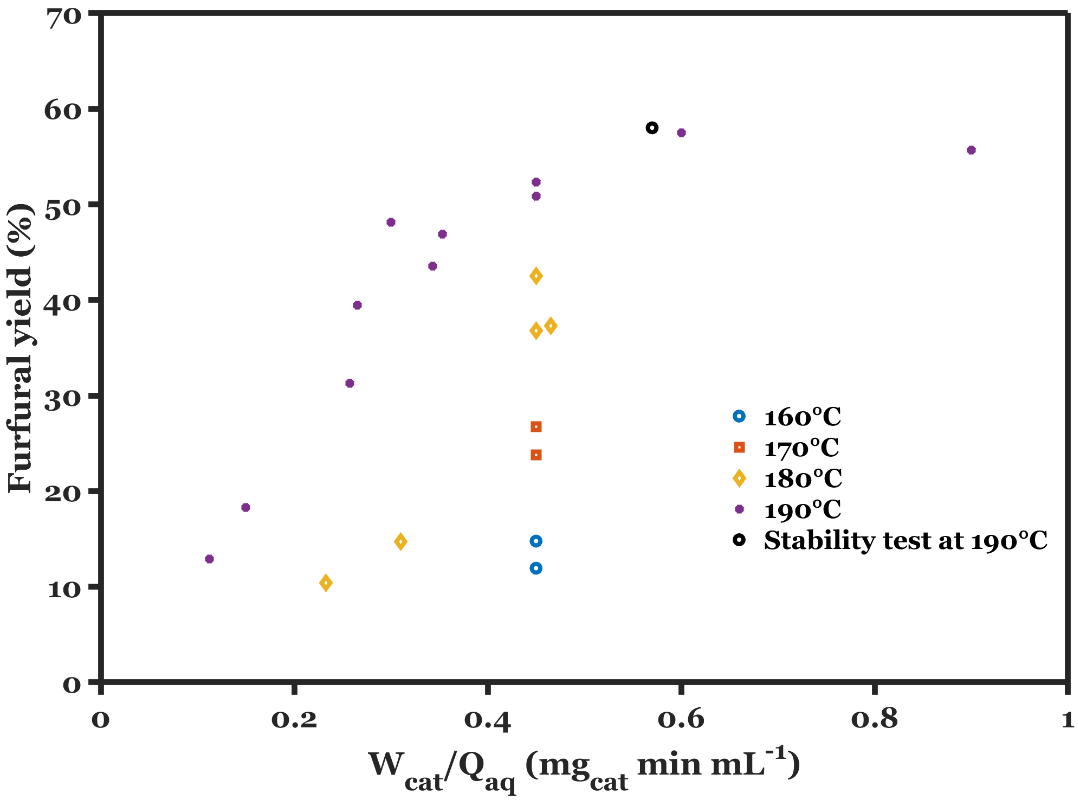

Figure S14: Furfural yield *vs.* normalized residence time (expressed as $mg_{cat}\,min\,mL^{-1}$).

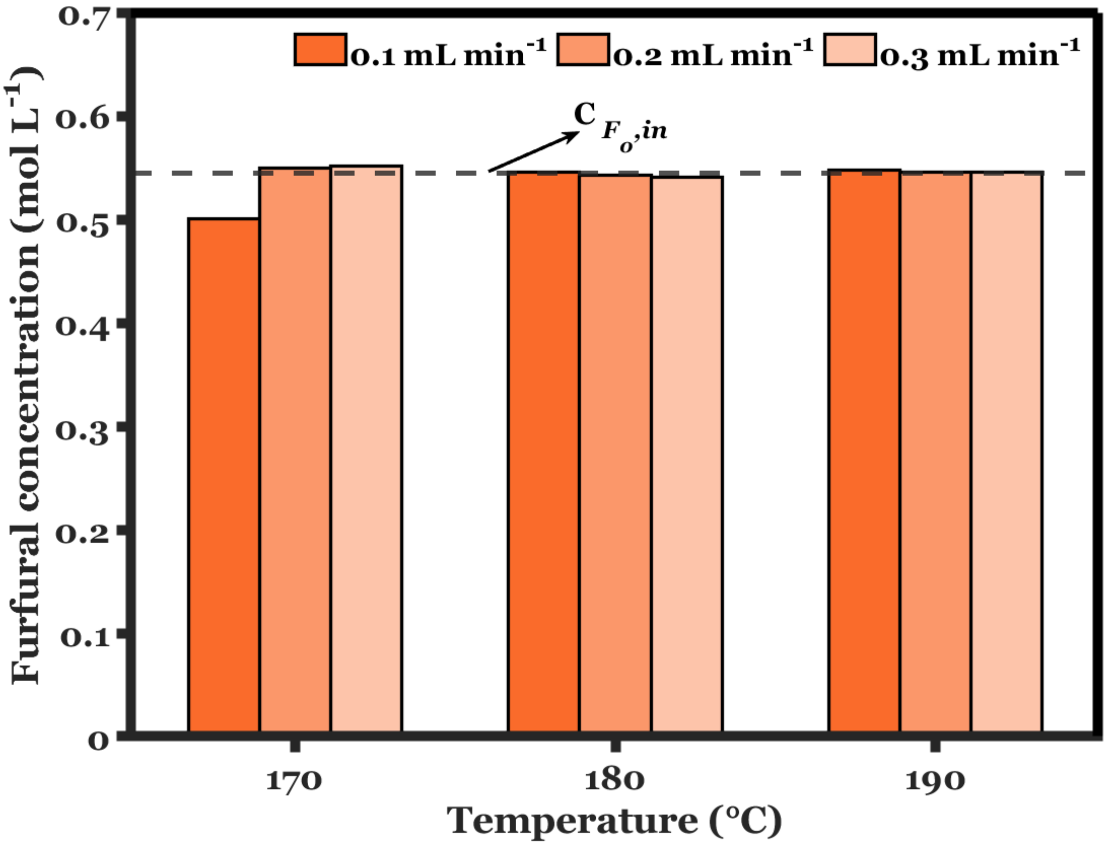

Figure S15: Effect of flow rate and temperature on furfural degradation in flow reactor with SBP. Reaction conditions: T = 170-190°C; Foam density = 40 PPI; $C_{furfural_0,aq}$ = 3 wt.%; $Q_{toluene}$:Q_{aq} = 2:1 (vol.:vol.); W_{cat} = 90 mg; Catalyst = TiO_2.

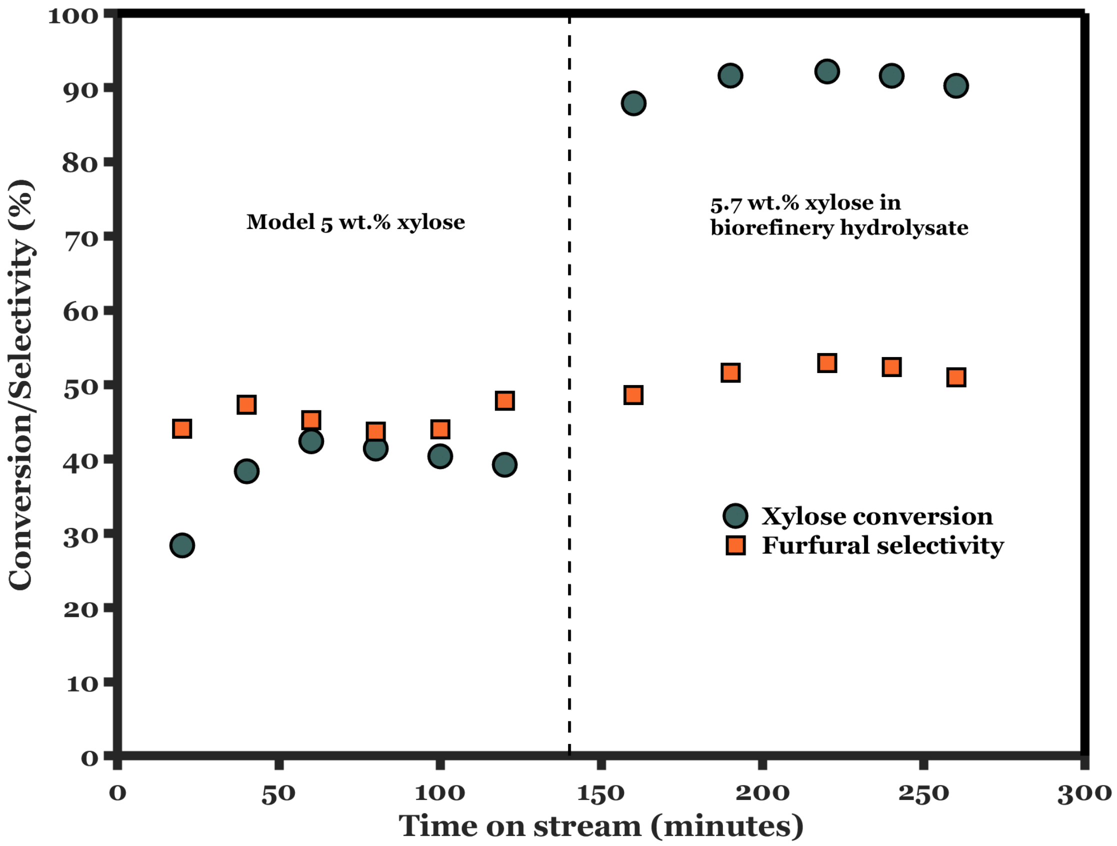

Figure S16: Xylose conversion and furfural selectivity using model xylose and biorefinery hydrolysate over TiO_2 coated foam catalysts. Reaction conditions : Temperature = 180°C; W_{cat} = 90 mg; Foam density = 40 PPI; Q_{aq} = 0.2 mL min^{-1}; Q_{SBP}:Q_{aq} = 2:1 (vol.:vol.).

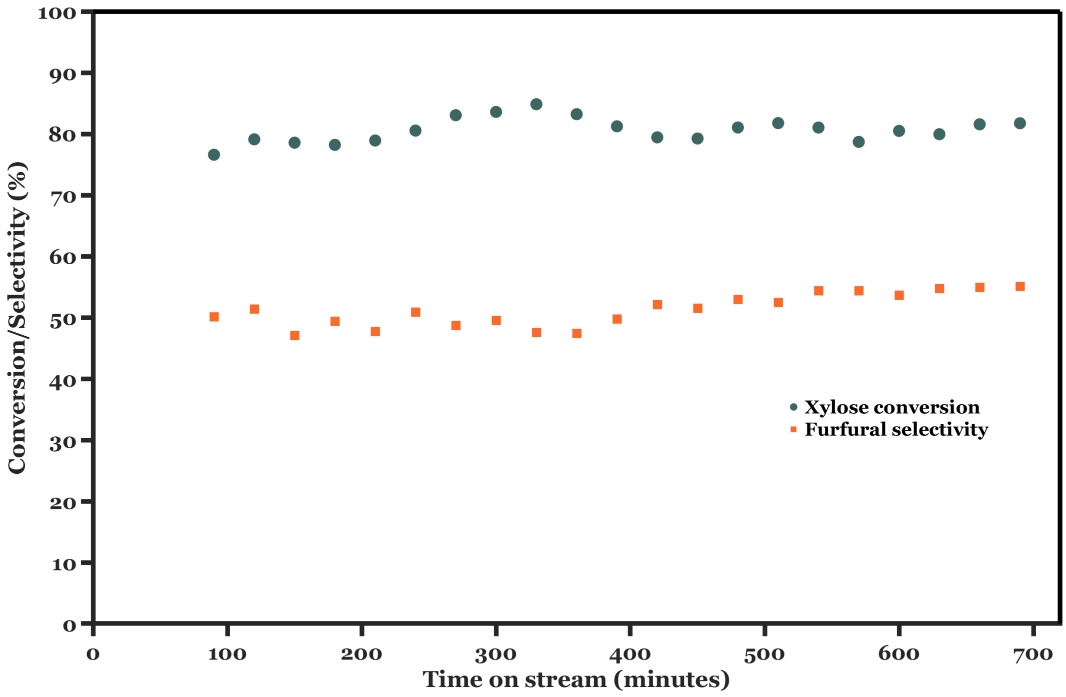

Figure S17: Xylose conversion and furfural selectivity using biorefinery feed over TiO_2 coated foam catalysts. Reaction conditions : Temperature = 180°C; W_{cat} = 90 mg; Foam density = 40 PPI; $Q_{aq} = 0.2$ mL min^{-1}; $Q_{SBP} = 0.4$ mL min^{-1}; $C_{X_0,aq} = 5.7$ wt.%

4.3 Autoclave experiments

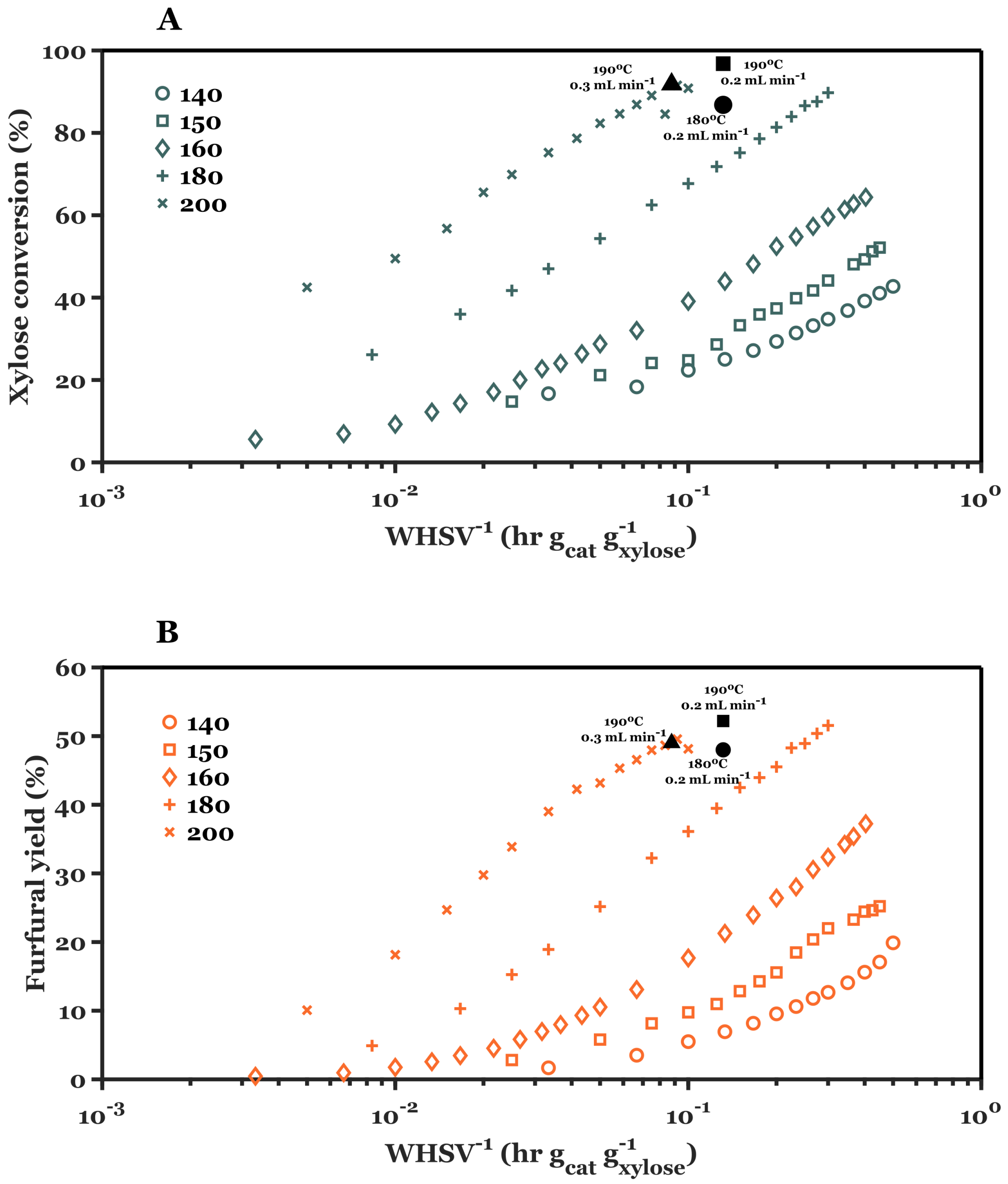

Figure S18: Comparison of (A) - xylose conversion, and (B) - furfural yield obtained with biphasic flow reactor *vs.* autoclave with monophasic conditions. The colored open symbols indicate autoclave experiments while the closed black colored markers indicate biphasic flow experiments. NOTE: The numbers in legend indicate different operating temperatures (°C) for autoclave experiments under monophasic conditions. Reaction conditions for autoclave experiments : T = 140 to 200°C; Reaction time = 60 to 180 minutes; Stirrer speed = 600 RPM; Catalyst = TiO_2; W_{cat} = 800 mg; xylose : catalyst ratio = 10:1 mass basis. Reaction conditions for flow experiments : W_{cat} = 90 mg; Foam density = 40 PPI; Q_{aq} = 0.2 $mL\,min^{-1}$; Markers with figures stating temperature and aqueous flow rate indicate results for flow experiments.

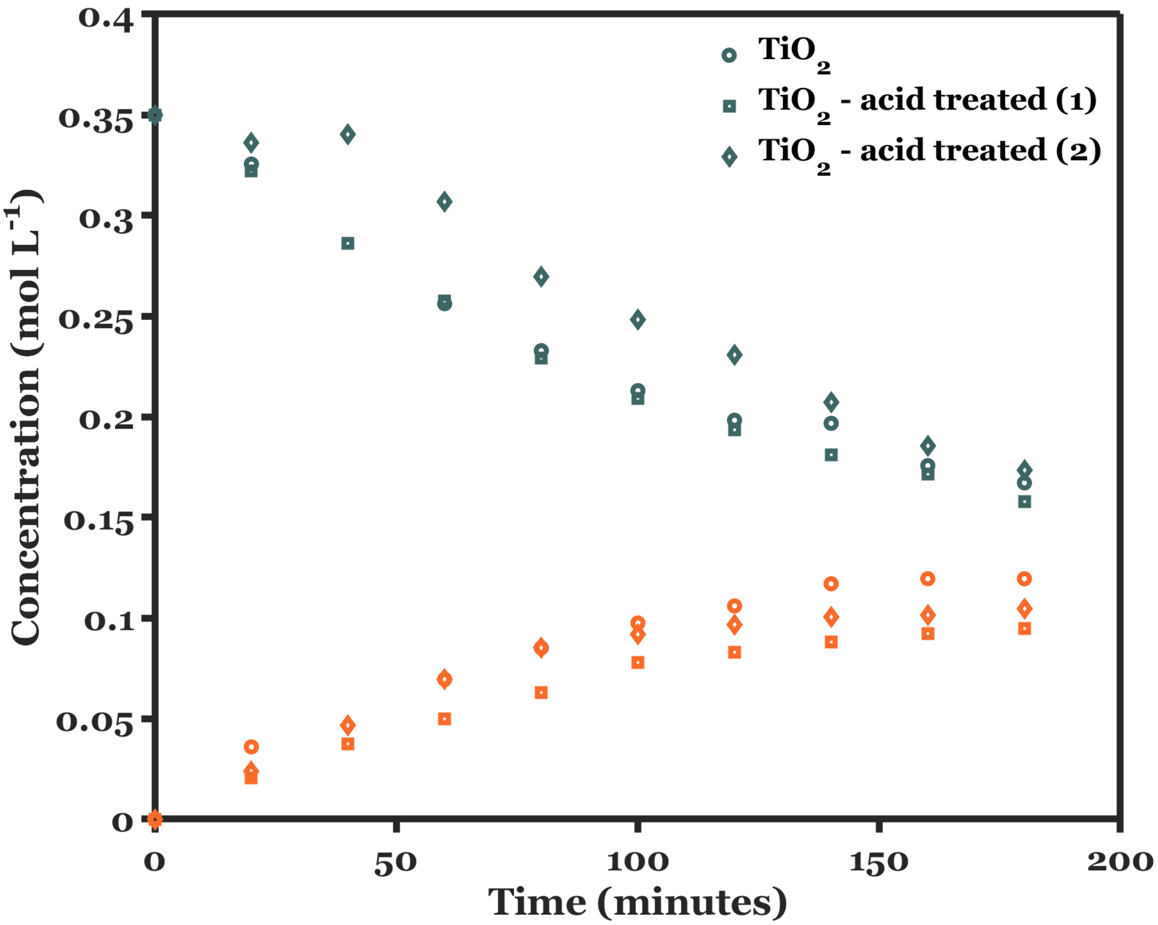

Figure S19: Comparison of xylose and furfural concentration with reaction time for commercial TiO_2 and HCl treated TiO_2 under monophasic conditions in an autoclave. Green and orange markers indicate xylose and furfural, respectively. Reaction conditions : Temperature = 160°C; $C_{X_0,aq}$ = 5 wt.%; Stirrer speed = 600 RPM; Reaction time = 3 hours; Catalyst = TiO_2; Catalyst : xylose = 1:10 mass basis.

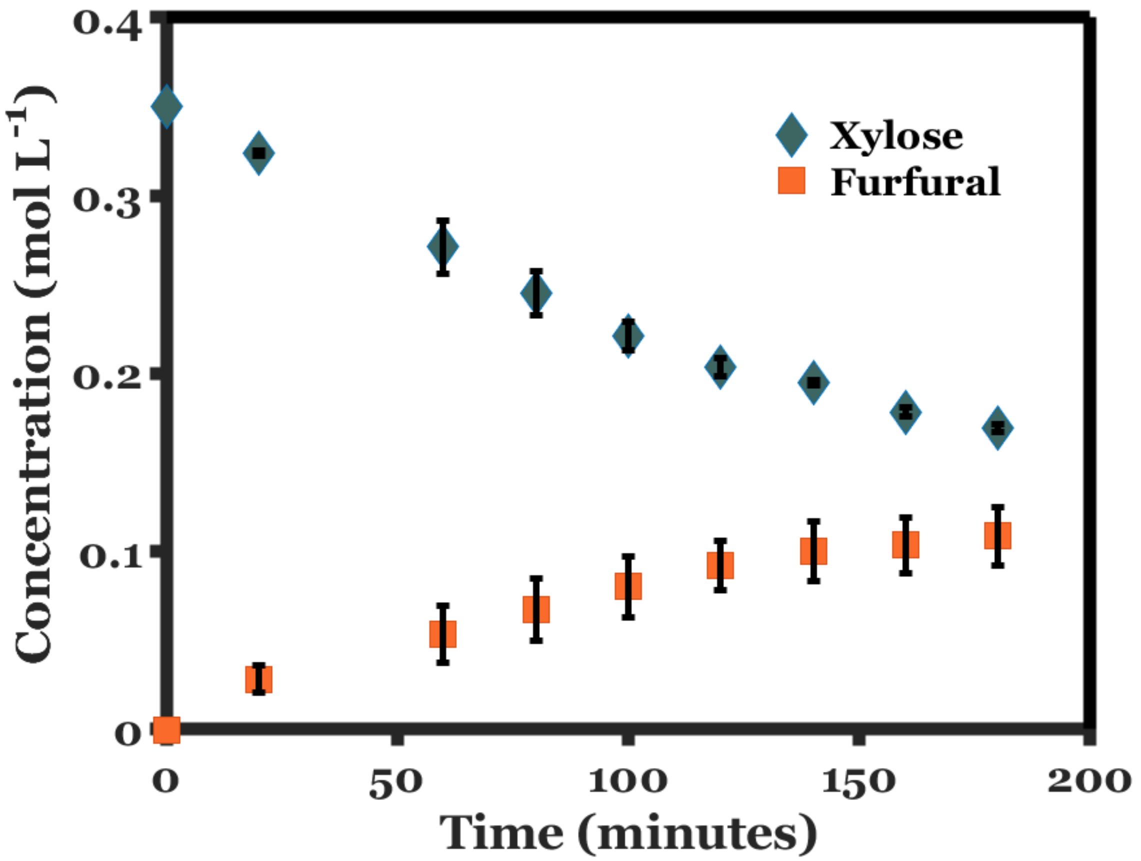

Figure S20: Xylose conversion to furfural in monophasic conditions using an autoclave reactor. Reaction conditions: T = 160°C; $C_{X_0,aq}$ = 5 wt.%; Stirrer speed = 600 RPM; Reaction time = 3 hours; Catalyst = TiO_2; Catalyst : xylose = 1:10 mass basis.

4.4 GCMS analysis of reaction samples

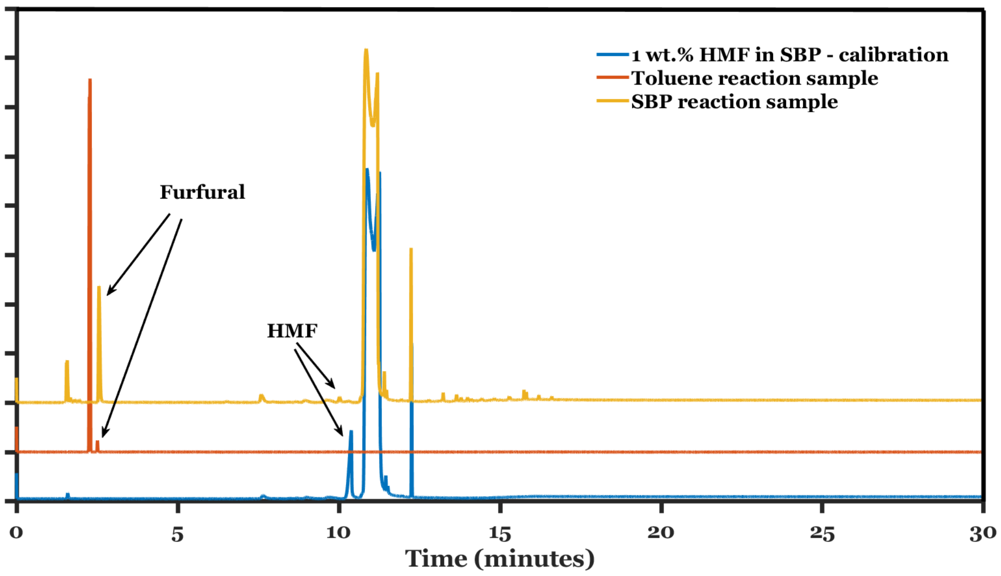

Figure S21: GCMS chromatogram of reaction samples with toluene and SBP as the organic solvent.

5 Partitioning experiments with organic solvents

The definition of furfural partition coefficient for a given organic solvent is calculated according to the below stated equation:

$$m_{furfural} = \frac{C_{furfural,organicphase}}{C_{furfural,aqueousphase}} \tag{S10}$$

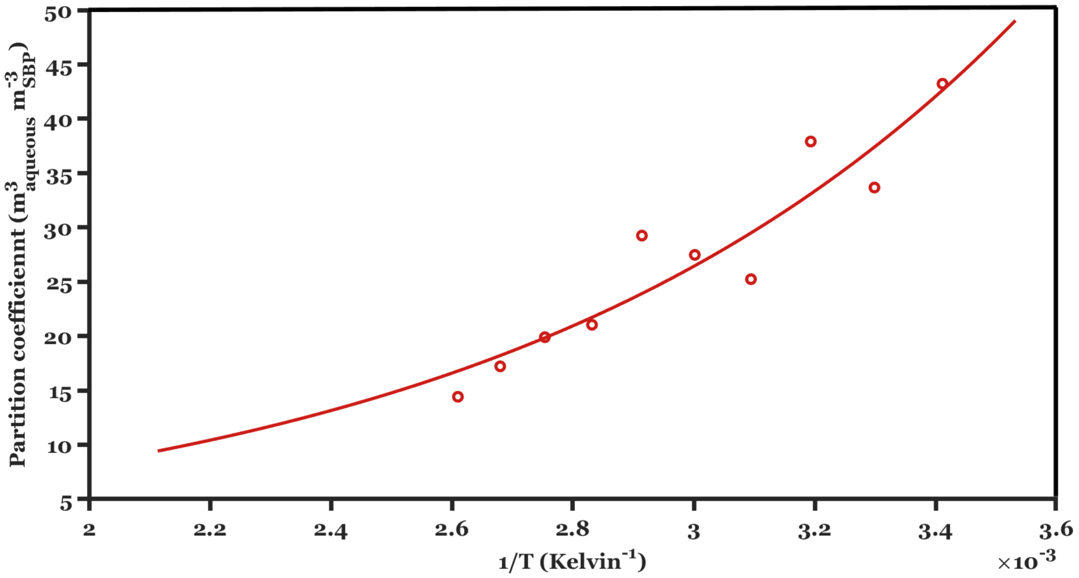

Figure S22: Partition coefficient variation for SBP with temperature. A 1:1 volumetric ratio of SBP : aqueous (3 wt.%) furfural solution was used for all experiments. Temperature = 20-100°C. The solid line indicates fitting result.

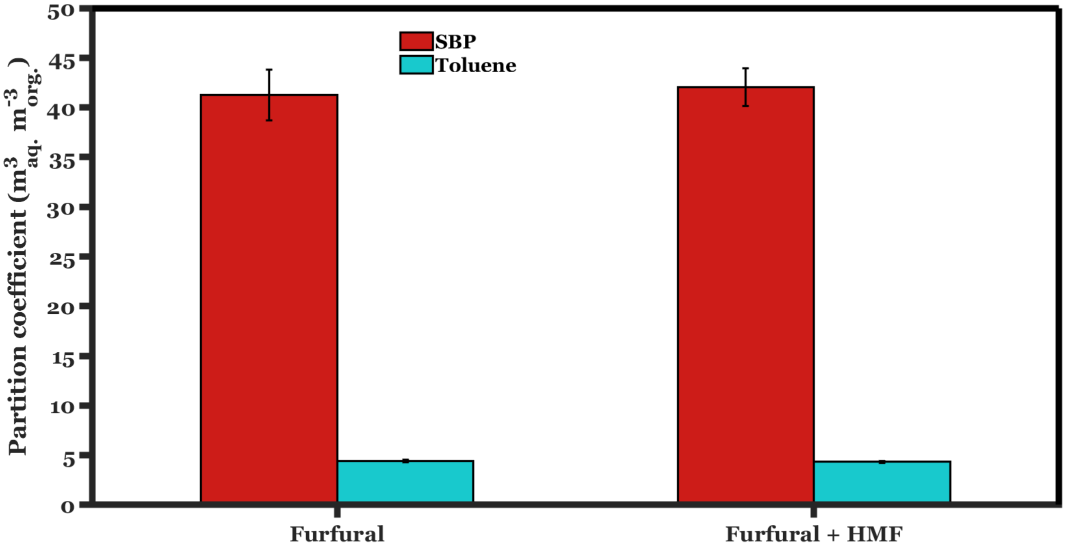

Figure S23: Partition coefficient for toluene and SBP with furfural and a mixture of furfural and HMF. A 1:1 volumetric ratio of organic solvent : aqueous solution was used. A 3 wt.% furfural solution and a mix of 3 and 0.5 wt.% HMF solution was used. Temperature = 30°C. The experiments were performed in duplos.

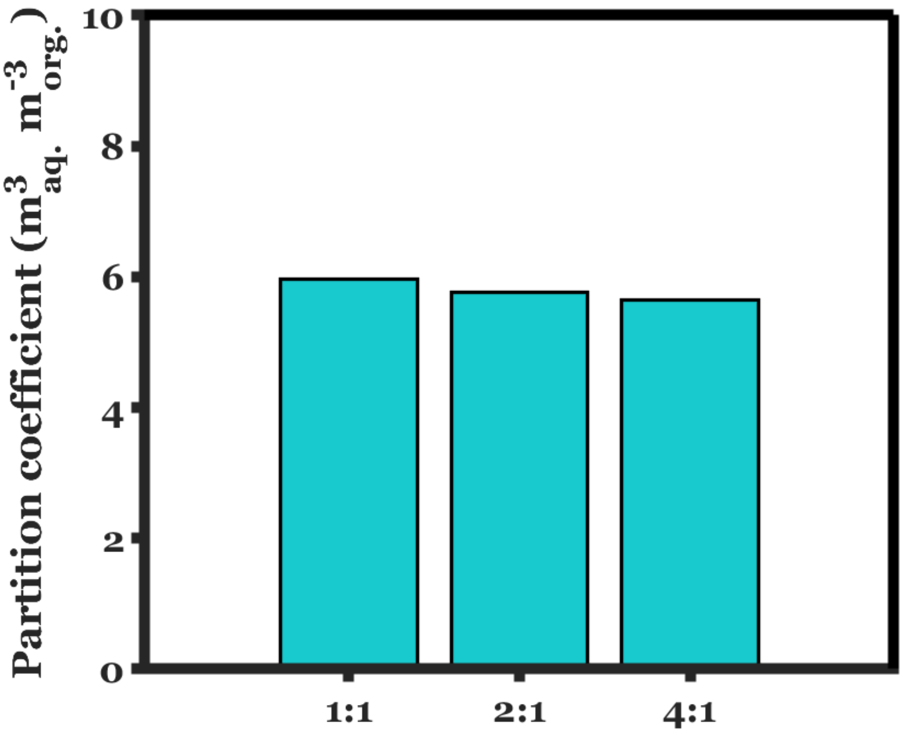

Figure S24: Partition coefficient for SBP with HMF. Different volumetric ratio of organic solvent : aqueous solution were used as indicated by the labels on X- axis. A 1 wt.% HMF aqueous solution was used. Temperature = 30°C.

6 Mass transfer time scales

This section describes the approximation of both liquid-liquid and solid-liquid mass transfer rates involving furfural formed during xylose dehydration. To prevent furfural degradation, the time scales for furfural extraction from aqueous phase should be smaller than its degradation rate in the 33 mM H_2SO_4 aqueous environment and its synthesis rate (approximated by calculation of k_i (see Figure S25).

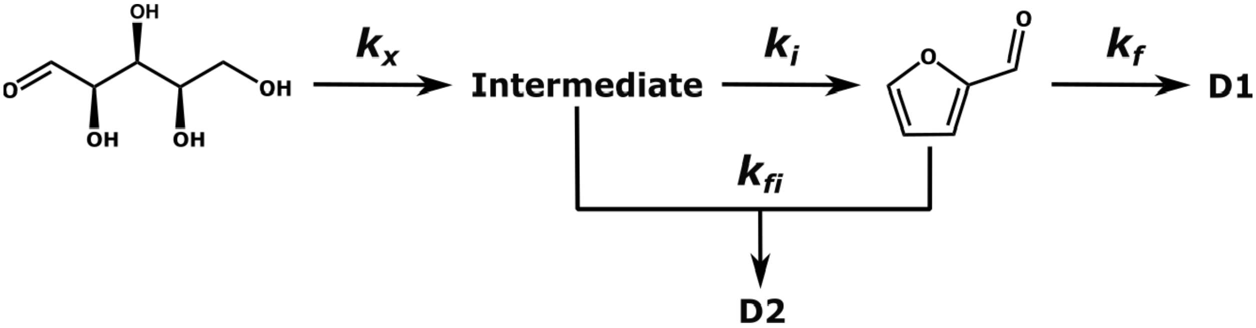

Figure S25: Reaction mechanism for xylose dehydration to furfural.

6.1 Liquid-liquid extraction

Liquid-liquid mass transfer rates can be obtained based on correlations for trickle flow packed bed configuration[12] as follows:

$$k_C A = 0.06 \epsilon_D \epsilon_C \left(\frac{A_p \rho_C (1 - \epsilon_B)}{g \Delta \rho} \right)^{-0.5} \left(\frac{\mu}{\rho D} \right)^{-0.5} \left(\frac{\sigma}{\Delta \rho g} \right)^{-0.5} \tag{S11}$$

where k_C A is the volumetric mass transfer rate for liquid-liquid extraction of the solute from the dispersed to continuous phase, ϵ is the hold-up (subscripts B, C and D denote solid, continuous and dispersed phase hold-ups respectively), σ is the surface tension for dispersed phase, μ is viscosity of water, ρ is density of liquids and D is diffusion coefficient of furfural. Substituting the respective numerical values of the variables, a volumetric mass transfer rate of $2.2 \cdot 10^{-4}$ s^{-1} is obtained. However, such correlations for liquid-liquid extraction using foam structures cannot be found in the current existing literature.

6.2 Solid-liquid mass transfer

Solid-liquid mass transfer rates can be obtained based on correlations for trickle flow configuration with foam packing[13] as follows:

$$Sh = 0.13 Re_L^{0.805} Re_G^{-0.89} \left(a_s d_w \frac{1-\epsilon}{\epsilon} \right)^{-1.34} Sc^{1/3} \tag{S12}$$

where Sh is the Sherwood number, Re is Reynolds number, Sc is Schmidt number, a_s is interfacial area, d_w is the window diameter of foams and ϵ is solid hold up. Substituting the numerical values in the expression, an overall volumetric mass transfer rate of $7.6 \cdot 10^{-3}$ s^{-1} is obtained.

6.3 Furfural degradation and formation rates in 33 mM H_2SO_4 and TiO_2

Using the pre-exponential factors and activation energies (listed in Table S4) from the work of Papaioannou et al.,[14] furfural formation rate of $8.0 \cdot 10^{-3}$ s^{-1} is catalyzed by H_2SO_4; in addition, furfural degrades at $9.2 \cdot 10^{-5}$ s^{-1} at 190°C. Similarly, TiO_2 catalyzes furfural formation at $1.46 \cdot 10^{-2}$ s^{-1}, while degradation of furfural occurs at $1.0 \cdot 10^{-4}$ s^{-1} over the solid catalyst.

Table S4: Kinetic parameters for furfural formation and degradation in presence of H_2SO_4, based on the work of Papaioannou et al.[14]

	k_x	k_i	k_f
Pre-exponential factor (s^{-1})	exp(38.6)	exp(34.89)	exp(8.57)
Activation energy ($kJ\,mol^{-1}$)	159.1	136.2	62

From the above listed calculations in subsections 6.1., 6.2. and 6.3., the mass transfer rates for furfural removal from the solid catalyst surface to aqueous phase and its extraction into the organic phase are significantly greater than its degradation but similar to its formation rates. Hence, the process is reaction-limited in our work.

6.4 Calculation of kinetic parameters for TiO_2

Based on the batch experiments performed in this work, shown in Figure S18, respective pre-exponential factors and activation energy for each reaction step were determined. The contribution of catalytic activity due to high temperature liquid water for xylose dehydration was determined based on experimental data from Jakob et al.[15]

For the flow experiments performed in this work, xylose conversion remained unchanged at different concentrations. Hence, the assumption of first-order behaviour with respect to xylose is valid. The relationship between conversion (X_a) and the rate constant at a given temperature can be expressed as shown in equation S13.

$$ln(1 - X_a) = k_{overall} \cdot \tau_L \tag{S13}$$

where τ_L is the liquid residence time in the flow reactor, 2.6 minutes in our case.

For the experiments reported in Figure 3 of the main manuscript, the liquid flow rates were kept constant. Hence, ln(1-X_a) is proportional to the rate constant ($k_{overall}$) at a given temperature.

Similarly, based on the kinetic constants for xylose conversion (k_x, as listed in Table 4) and the residence time, contribution of H_2SO_4 towards xylose conversion can be calculated. This can then be subtracted from the overall conversion to obtain xylose conversion using TiO_2 as the catalyst.

Since the rate constant follows Arrhenius relationship with temperature, the conversion obtained at different operating temperature (as shown in Figure 3 of the main manuscript) can be used to obtain activation energy for xylose conversion. This is shown as the slope of the fitted line obtained in Figure 9 of the main manuscript.

The activation energies obtained for the flow and batch reactor configuration are similar to each other, i.e., 91.6 and 86 $kJ\,mol^{-1}$ respectively.

References

(1) Gómez Millán, G.; Ashok, B.; Prasad, R.; Oinas, P.; Llorca, J.; Sixta, H. Furfural production from xylose and birch hydrolysate liquor in a biphasic system and techno-economic analysis. *Biomass Conversion and Biorefinery* **2021**, *11*, 2095–2106.

(2) Deng, A.; Lin, Q.; Yan, Y.; Li, H.; Ren, J.; Liu, C.; Sun, R. A feasible process for furfural production from the pre-hydrolysis liquor of corncob via biochar catalysts in a new biphasic system. *Bioresource Technology* **2016**, *216*, 754–760.

(3) Mittal, A.; Black, S. K.; Vinzant, T. B.; O'Brien, M.; Tucker, M. P.; Johnson, D. K. Production of Furfural from Process-Relevant Biomass-Derived Pentoses in a Biphasic Reaction System. *ACS Sustainable Chemistry & Engineering* **2017**, *5*, 5694–5701.

(4) Liu, J.; Liu, H.; Chen, L.; An, Y.; Jin, X.; Li, X.; Liu, Z.; Wang, G.; Liu, R. Study on the removal of lignin from pre-hydrolysis liquor by laccase-induced polymerization and the conversion of xylose to furfural. *Green Chemistry* **2022**, *24*, 1603–1614.

(5) Liu, H.; Hu, H.; Jahan, M. S.; Ni, Y. Furfural formation from the pre-hydrolysis liquor of a hardwood kraft-based dissolving pulp production process. *Bioresource Technology* **2013**, *131*, 315–320.

(6) Liu, H.; Hu, H.; Baktash, M. M.; Jahan, M. S.; Ahsan, L.; Ni, Y. Kinetics of furfural production from pre-hydrolysis liquor (PHL) of a kraft-based hardwood dissolving pulp production process. *Biomass and Bioenergy* **2014**, *66*, 320–327.

(7) Shen, T.; Hu, Y.; Hu, R.; Zhuang, W.; Li, M.; Niu, H.; Xu, H.; Zhu, C.; Ying, H. Continuous production of furfural from pulp prehydrolysate in a vaporization reactor. *Industrial Crops and Products* **2020**, *153*.

(8) Baktash, M. M.; Ahsan, L.; Ni, Y. Production of furfural from an industrial pre-hydrolysis liquor. *Separation and Purification Technology* **2015**, *149*, 407–412.

(9) Sheldon, R. A. Metrics of Green Chemistry and Sustainability: Past, Present, and Future. *ACS Sustainable Chemistry & Engineering* **2018**, *6*, 32–48.

(10) Brunauer, S.; Emmett, P. H.; Teller, E. Adsorption of gases in multimolecular layers. *Journal of the American chemical society* **1938**, *60*, 309–319.

(11) Barrett, E. P.; Joyner, L. G.; Halenda, P. P. The determination of pore volume and area distributions in porous substances. I. Computations from nitrogen isotherms. *Journal of the American Chemical Society* **1951**, *73*, 373–380.

(12) Trambouze, P.; Landeghem, H. v.; Wauquier, J.-P. *Chemical Reactors: Design, Engineering, Operation*; Editions Technip, 1988; pp 299–346.

(13) Mohammed, I.; Bauer, T.; Schubert, M.; Lange, R. Liquid–solid mass transfer in a tubular reactor with solid foam packings. *Chemical Engineering Science* **2014**, *108*, 223–232.

(14) Papaioannou, M. Biphasic Continuous Synthesis of Furfural: Insights into Reactor and Process Optimization. Phd Thesis 1 (Research TU/e / Graduation TU/e), Chemical Engineering and Chemistry, 2023; Proefschrift.

(15) Jakob, A.; Likozar, B.; Grilc, M. Aqueous conversion of monosaccharides to furans: were we wrong all along to use catalysts? *Green Chemistry* **2022**, *24*, 8523–8537.
